# Biomimetic Atorvastatin Self-Assembled Nanomedicine Inhibits the Cyclooxygenase-2/Prostaglandin E2 Pathway Enhanced Photothermal and Antitumor Immunity

**DOI:** 10.34133/bmr.0149

**Published:** 2025-03-04

**Authors:** Min Zhou, Ruyue Han, Wenjie Xu, Xinyan Hao, Yanjin Peng, Yucheng Tang, Pengcheng Sun, Tiantian Tang, Junyong Wu, Daxiong Xiang

**Affiliations:** ^1^ Department of Pharmacy, The Second Xiangya Hospital, Central South University, Changsha 410011, China.; ^2^ Hunan Provincial Engineering Research Centre of Translational Medicine and Innovative Drug, Changsha 410011, China.; ^3^ Institute of Clinical Pharmacy, Central South University, Changsha 410011, China.; ^4^ Hunan Key Laboratory of Tumor Models and Individualized Medicine, The Second Xiangya Hospital, Central South University, Changsha 410011, China.

## Abstract

Cancer continues to pose remarkable medical challenges worldwide. While current cancer therapies can lead to initial clinical improvement, they are often followed by recurrence, metastasis, and drug resistance, underscoring the urgent need for innovative treatment strategies. Atorvastatin calcium (AC), a widely used lipid-lowering and anti-inflammation drug in the clinic, has shown antitumor potential. To further improve the antitumor efficacy, we developed self-assembled AC and polydopamine (PDA) nanoparticles whose surface was coated with macrophage membranes (CM) as a biomimetic drug delivery system [AC@PDA@CM (APM)]. APM showed high drug-loading capacity, excellent stability, excellent bioavailability, and tumor-targeting ability, ultimately achieving photothermal synergistic cancer immunotherapy. Our findings indicate that APM efficiently delivers AC to tumor sites while leveraging photothermal therapy (PTT) to enhance local tumor ablation and antitumor immune effect. Notably, APM mitigates tumor immunosuppression triggered by PTT through AC, suppressing the COX-2/PGE2 pathway and immune evasion signal CD47. Furthermore, APM notably reduced nonspecific distribution and side effects, which is conducive to ensuring the safety level of medication. This integrated approach boosts therapeutic efficacy and highlights the potential of APM as a multifunctional agent for cancer therapy, paving the way for future clinical applications.

## Introduction

Cancer remains one of the most serious global health challenges [1]. While emerging targeted therapies and immunotherapies have shown crucial promise by offering higher efficacy, reduced toxicity, and prolonged survival [2], their effectiveness is often limited by drug resistance [3]. In recent years, drug repurposing has gained great interest as a feasible strategy for accelerating anticancer candidate development using clinically approved drugs. Notably, statins, widely used 3-hydroxy-3-methylglutaryl coenzyme A (HMG-CoA) reductase inhibitors for lipid-lowering and anti-inflammatory purposes, have shown anticancer potentials [4]. For example, atorvastatin calcium (AC) has been shown to reduce cholesterol levels in mitochondrial membranes in liver cancer, thereby enhancing the efficacy of chemotherapy [5]. Besides, AC not only restrains the proliferation of glioblastoma (GBM) and devil facial tumor disease (DFTD) through the inhibition of the mevalonate pathway [6,7] but also suppresses the metastasis of head and neck cancer as well as melanoma [8]. Moreover, AC mediates immune mechanisms by promoting fatty acid oxidation, increasing reactive oxygen species (ROS) generation, and inducing immunogenic cell death (ICD) [9]. These processes further enhance the release of tumor antigens and damage-associated molecular patterns (DAMPs) to activate dendritic cells (DCs) and cytotoxic T lymphocytes (CTLs), leading to robust antitumor immune responses [10,11]. Furthermore, statins have been found to downgrade the expression of integrin-associated protein [cluster of differentiation 47 (CD47)] [12], a key player in immune evasion, by disrupting the “do not eat me” signal to macrophage-mediated phagocytosis in atherosclerosis therapy [13,14]. This suggests that AC has the potential to suppress the immune escape mechanisms in cancer through a similar mechanism. Despite these promising antitumor properties, the application of free AC is hindered by the limited bioavailability due to its poor water solubility [15–17]. Existing nanoformulations like micelles and liposomes have low drug-loading capacities [4,18,19], requiring large amounts of material to achieve therapeutic doses, which could pose safety concerns. This underscores the need for developing innovative drug delivery systems to enhance drug loading and improve the therapeutic efficacy of AC.

Given the amphiphilic nature of AC (hydrophilic carboxylic acid and hydrophobic phenyl rings), we explored, in the aqueous phase, the formation of self-assembled AC nanoparticles, which exhibited poor stability. To improve the stability, we employed polydopamine (PDA), which has excellent biocompatibility and surface modifiability [20,21], to form AC@PDA (AP), which showed stability for up to 14 d. More importantly, PDA possesses photothermal capability [22–24], which can be used for antitumor photothermal therapy (PTT). On the other hand, AC is known for its anti-inflammatory properties [25], which are expected to inhibit the cyclooxygenase-2 (COX-2)/prostaglandin E2 (PGE2) pathway to alleviate the immunosuppressive tumor environment [26], which is often exacerbated after PTT because of local inflammation-induced excessive expression of COX-2 and the accumulation of the downstream lipid metabolite PGE2 in the tumor [27,28]. This hybrid nanoparticle not only has improved stability but also strengthens the PTT in a synergistic approach [29]. Further, we modified the nanoparticle by macrophage membrane (CM) coating on the surface to improve in vivo stability and enhance the tumor-targeting ability with biointerfacing to enhance the antitumor therapy [30,31].

Here, we present a new strategy for delivering therapeutic self-assembled AP nanoparticles with CM coating for tumor therapy. Briefly, this AC@PDA@CM (APM) nanoformulation is expected to safely achieve synergistic metabolic therapy and PTT with enhanced antitumor immune response. We believe that this nanoplatform holds great promise as a comprehensive strategy for cancer therapy with prominent translational potential.

## Materials and Methods

### Chemicals and reagents

AC was obtained from Bide Pharm (Shanghai, China). Dopamine hydrochloride (PDA) was obtained from Aladdin Biochemical Technology (Shanghai, China). Tris (trimethyl siloxy) ethylene (Tris) was obtained from VETEC (USA). Dimethyl sulfoxide (DMSO) was obtained from Sinopharm Chemical Reagent Co. Ltd. (China). Cell Counting Kit-8 (CCK-8) was obtained from NCM Biotech (Suzhou, China). Cell Membrane Green Fluorescent Probe DiO (DiO) was obtained from Beyotime Biotechnology (China). Cell Membrane Orange-Red Fluorescent Probe DiR (DiR) was obtained from Yeasen Biotechnology (China). ROS Analysis Kit (HDCF-DA) was obtained from KeyGEN BioTECH (Jiangsu, China). 4′,6-Diamidino-2-phenylindole (DAPI) and phosphate-buffered saline (PBS) were obtained from Servicebio (Wuhan, China). Calcein AM/propidium iodide (PI) double staining kit was obtained from Maokang Biotechnology Co. Ltd. (Shanghai, China). Rabbit antibodies including HMGB1, CRT, CD47, and GAPDH were obtained from Abcam (UK), and COX-2 was obtained from Abclonal (USA). Annexin V-FITC/PI Cell Apoptosis Detection Double Staining Kit and antibodies used for flow cytometry (FCM) were obtained from BD Pharmingen (USA). Mouse PGE2 ELISA (enzyme-linked immunosorbent assay) kit was obtained from Bioswamp (Wuhan, China). Antibiotics were obtained from Beijing Solarbio Science & Technology Co. Ltd. (Beijing, China). Sodium pyruvate and glucose were obtained from Invitrogen (USA). Fetal bovine serum (FBS), RPMI 1640 medium, and Dulbecco’s modified Eagle’s minimum essential medium (DMEM) were obtained from VivaCell (Shanghai, China).

### Cell culture and animals

4T1 mouse mammary carcinoma cell line was cultured in RPMI 1640 medium, supplemented with 1% GlutaMAX, 1% sodium pyruvate, and 10% FBS. RAW 264.7 macrophage cell line was cultured in DMEM supplemented with 10% FBS. Incubation conditions for both cell types were set at 37 °C within a 5% CO_2_ atmosphere in a humidified incubator. Female BALB/c mice, aged 6 to 8 weeks, were obtained from the SJA Laboratory Animal Co. Ltd. (Changsha, China). Animals were housed in accordance with the Institutional Animal Care and Use Committee (IACUC) protocols. All animal experiments adhered to ethical guidelines and were approved by the IACUCs of the Department of Laboratory Animals, Second Xiangya Hospital, Central South University.

### Isolation of RAW 264.7 macrophage membranes

The preparation of isolation buffer 1 (IB-1) and isolation buffer 2 (IB-2) followed the methods reported by Suski et al. [32]. RAW 264.7 macrophage cells were cultured in 100-mm dishes until they reached 90% to 100% confluence. Cells were then harvested using a spatula, centrifuged at 1,000 rpm for 5 min, and resuspended in IB-1. The cells were lysed on ice for 15 min using an ultrasonic cell grinder (SCIENTZ-48, China). The lysate was first centrifuged at 800*g* for 10 min at 4 °C (Neofuge 15R, Heal Force, China). The supernatant was centrifugated at 10,000*g* for 10 min at 4 °C (Neofuge 15R, Heal Force, China). The supernatant was further ultracentrifuged at 100,000*g* for 1 h at 4 °C (Optima XPN, Beckman, USA). The membrane fraction as the pellet was resuspended in IB-2 and stored at −80 °C.

### Preparation and characterization of APM

First, appropriate amounts of AC, PDA, and tris (hydroxymethyl) aminomethane (Tris) were weighed separately. Then, AC was added to DMSO. Separately, PDA and Tris were dissolved in deionized water. Next, each of them was sonicated for 5 min (KQ218, Kun Shan Ultrasonic Instruments, China) to obtain 10 mg/ml AC, 10 mg/ml PDA, and 6 mg/ml Tris solutions. The AC solution (60 μl) was slowly added dropwise to 2 ml of deionized water while maintaining constant stirring at 25 °C over 5 min. The PDA solution (30 μl) and the Tris solution (30 μl) were then introduced into the above solution. The resulting mixture was left in the dark at 25 °C for 24 h and was placed into a dialysis bag with a molecular weight cutoff (MWCO) of 10 kDa. PBS was used as the dialysis solvent for 24 h to remove free AC and PDA, resulting in the purified AP solution. The AP (300 μg/ml) was then mixed with CM (1 mg/ml) at a 4:1 ratio of volume and exposed to sonicate for 5 min in the dark, followed by transfer into a dialysis bag (10 kDa). PBS was used as the dialysis solvent over 24 h to remove unbound AP and CM obtaining the purified APM formulation, which was stored at 4 °C in the dark.

The size distribution, polydisperse index (PDI), and Zeta potential of AP and APM were analyzed by dynamic light scattering (DLS) (Zetasizer Nano ZS90, Malvern, UK) with deionized water as the solvent. Morphology images of AP and APM were captured by transmission electron microscopy (TEM) (Tecnai G2 Spirit Twin, FEI, Holland). Protein profiles of APM were obtained using Coomassie Brilliant Blue staining (InstantBlue, Abcam, UK) and a gel imaging system (ChemiDoc Touch, Bio-Rad, USA). The encapsulation efficiency of CM in APM was determined by nano-flow cytometry. For the sample, the CM was labeled by mixing with DiO fluorescent dye (1 mM) at a volume ratio of 20:1 and incubated at 37 °C for 15 min. Subsequent centrifugation with 12,000 rpm for 5 min (Heraeus Fresco 17, Thermo Fisher Scientific, Germany) was employed to remove the supernatant containing unbound DiO. The DiO-labeled CM was then used to encapsulate the AP to form the APM. The nano-flow cytometer (Flow NanoAnalyzer Fuliu Biotechnology, China) is equipped with a 488-nm laser. The instrument was calibrated using 250-nm silica beads at a defined concentration of 2.14 × 10^10^ particles/ml, which served as the reference for particle concentration. Monodispersed silica beads with 4 different diameters, including 68, 91, 113, and 155 nm, were utilized as size reference standards. Measurements of freshly filtered (0.1 μm) PBS were taken as background signals. The instrument detected forward scatter (FSC) and side scatter (SSC) signals for size and concentration analysis for 1 min under a sample pressure of 1.0 kPa, which measured the fluorescence signal of DiO to quantify CM encapsulation efficiency on APM by NanoFCM software (NF Prolession V2.0). Ultraviolet–visible (UV–Vis) absorbance spectra of PDA, AC, AP, and APM were measured using a multimode microplate reader (Varioskan LUX, Thermo Scientific, USA). Additionally, measurements were also conducted in sodium chloride (NaCl) solution, as well as in urea and sodium dodecyl sulfate (SDS) solutions, to evaluate their effects on the absorbance characteristics of AC, AP, and APM.

### Photothermal effect and photothermal stability of APM

The photothermal effect of APM formed with varying concentrations of PDA (0, 50, 100, 150, and 200 μg/ml) was evaluated by irradiating 1 ml of various APM with an 808-nm near-infrared (NIR) laser (1 W/cm^2^, 10 min) (FC-808 fiber-coupled laser system, CNI Photoelectric Technology, China). Additionally, the impact of various concentrations of APM (0, 50, 100, 150, and 200 μg/ml) and different laser powers (0.5, 1.0, 1.5, and 2.0 W/cm^2^) on the photothermal effect was also examined. To assess photothermal stability, 1 ml of APM (200 μg/ml) was irradiated with 1 W/cm^2^ NIR for 10 min and cooled to room temperature, and the cycle was repeated 4 times. Temperature changes were recorded every 30 s using a thermal camera (FLIR C5, FLIR Systems, USA). To evaluate the photothermal conversion efficiency of APM, a curve depicting the variation of temperature concerning time was recorded when 1 ml of APM solution (200 μg/ml) was heated to the equilibrium temperature under the 808-nm NIR (1 W/cm^2^) and then cooled down. Subsequently, the calculation followed the methods reported by Roper et al. [33]:

$$\eta =\frac{hS\left(T_{\mathrm{Max}}-T_{\text{Surr}}\right)-Q_{\mathrm{Dis}}}{I\left(1-10^{-A_{808\mathrm{nm}}}\right)}$$ (1)

where η represents photothermal conversion efficiency, h represents heat-transfer coefficient, ***ѕ*** represents the surface area of the container, $T_{\mathrm{Max}}$ represents the equilibrium temperature, $T_{\text{Surr}}$ represents the surrounding temperature, $Q_{\mathrm{Dis}}$ represents the heat dissipated from the photoabsorption of a quartz cuvette sample cell containing deionized water without nanoparticles, I represents the incident energy of the NIR laser (mW), and $A_{808\mathrm{nm}}$ represents the absorbance of the APM at a wavelength of 808-nm NIR,

$$\theta =\frac{T-T_{\text{Surr}}}{T_{\mathrm{Max}}-T_{\text{Surr}}}$$ (2)

$$\tau_{s}=\frac{\sum_{i}\;m_{i}\;C_{p,i}}{\mathrm{hS}}$$ (3)

$$t=-\;\tau_{s}\text{ln}\theta$$ (4)

where θ represents dimensionless driving force temperature, $\tau_{s}$ represents a time constant of the sample system, and $m_{i}$ and $\;C_{p,i}$ represent the mass and the specific heat capacity of the solvent (deionized water). Moreover, the thermal effects of PBS, AC, and APM (200 μg/ml) were compared under NIR irradiation (1 W/cm^2^, 10 min), with thermal images captured every 2 min.

### Drug loading and cumulative releases of APM

To quantify the drug loading capacity (DL) of APM nanoparticles, the purified APM solution was lyophilized using a vacuum freeze dryer (Lichen Bangxi Instrument Technology, China). The resulting mass of the nanoparticles was recorded as *M*_APM_. The content of AC in the nanoparticles (*M*_AC_) was then determined using high-performance liquid chromatography (HPLC) (LC-20A, SHIMADZU, Japan). The encapsulation efficiency (EE) was calculated based on the total amount of AC added (*M*_T_). The formulas are as follows:

$$DL\left(\%\right)=\frac{M_{\mathrm{AC}}}{M_{\mathrm{APM}}}\times 100\%$$ (5)

$$\mathrm{EE}\left(\%\right)=\frac{M_{\mathrm{AC}}}{M_{T}}\times 100\%$$ (6)

To evaluate the cumulative release profile of AC from the APM, we conducted in vitro release experiments using a dialysis device. A 10-ml aliquot of the purified APM solution was added to a dialysis bag with a MWCO of 10 kDa. The bag was then immersed in 200 ml of PBS at 37 °C under continuous stirring. At predetermined time points (0.5, 1, 2, 4, 6, 8, 10, 12, 24, and 48 h), samples were collected from the buffer. Fresh PBS was immediately added to maintain sink conditions. The concentration of AC in the samples was quantitatively determined using HPLC. To investigate the impact of NIR irradiation on AC release, a parallel assay was performed where NIR irradiation was applied to the dialysis setup at 12 h. The released AC was quantified by HPLC.

### Cellular uptake assay

The cellular uptake efficiency of the nanoparticles was evaluated using a fluorescence microscope (ECHO Revolve Microscope, APERBIO, USA). 4T1 cells were treated with DiO-labeled nanoparticles in 24-well plates containing coverslips for 2 h, followed by fixation with 4% paraformaldehyde for 15 min and staining of nuclei with DAPI for 10 min. Coverslips were mounted on slides with an antifade agent for microscopic observation. FCM was also performed to quantitatively analyze cellular uptake by measuring mean fluorescence intensity using a flow cytometer (NL3000, Cytek Bioscience, USA).

### In vitro cell viability assay

The viability of 4T1 cells exposed to various formulations was assessed using the CCK-8 assay. PDA, AC, AP, and APM were added in fresh RPMI 1640 medium, with AC concentrations of 0, 10, 20, 30, 40, 60, and 80 μg/ml, which were added to 96-well plates containing 4T1 cells at 70% confluence. After 12 h, cells in the groups requiring NIR treatment were irradiated for 10 min with 808-nm NIR (1.5 W/cm^2^). After an additional 12 h of incubation, cell viability was measured by CCK-8 assay using a microplate reader (INFINITE F50, TECAN, China).

### Live/dead cell staining and cell apoptosis evaluation

4T1 cells at 70% confluence in 24-well plates were incubated with blank RPMI 1640 medium (used as the control group), media containing PDA, AC, AP, or APM for 12 h. Cells in the groups requiring NIR treatment were irradiated with 808-nm NIR (1.5 W/cm^2^, 10 min). After a further 12-h incubation, live/dead cell staining was performed using a calcein AM/PI double staining kit. Fluorescence imaging was conducted with a 10× objective lens to assess cell viability. For apoptosis analysis, cells were treated consistently and stained with the Annexin V-FITC/PI Cell Apoptosis Detection Kit. Stained cells were analyzed by FCM.

### Cell migration and invasion evaluation

To assess cell migration, 4T1 cells were plated in 6-well plates with RPMI 1640 medium containing 10% FBS and incubated for 24 h. A scratch was made in the confluent cell monolayer using a sterile 200-μl pipette tip. Cells were washed 3 times with PBS to remove the floating cells. The cells were then incubated in a serum-free medium containing cholesterol, AC, AP, or APM. Wound closure was monitored by capturing images at 0 and 48 h after scratch using a fluorescent microscope (TH4-200, Olympus, Japan). For the invasion assay, transwell inserts were precoated with Matrigel. 4T1 cells suspended in 200 μl of serum-free RPMI 1640 medium were seeded onto the upper chambers. The lower chambers were added with 600 μl of RPMI 1640 medium with 15% FBS and different nanoparticles. After 48 h of incubation, the invading cells that adhered to the lower chamber were fixed with 4% paraformaldehyde and stained with 0.1% crystal violet solution. The stained cells were then imaged using an inverted microscope (TH4-200, Olympus, Japan) equipped with a 10× objective lens.

### ROS assay

4T1 cells were seeded in 24-well plates to achieve 70% confluence. These cells were incubated with blank RPMI 1640 medium (used as the control group), media containing PDA, AC, AP, or APM for 12 h. After washing twice with PBS for the NIR-treated cells, fresh RPMI 1640 medium was added before irradiation with 808-nm NIR (1.5 W/cm^2^, 10 min). Continue the incubation for 2 h, add the DCFH-DA probe, and incubate for another 30 min to label the fluorescence of ROS. Fluorescence was observed using an inverted microscope (TH4-200, Olympus, Japan) equipped with a 10× objective lens and quantified by FCM.

### Western blot and ELISA

When the 4T1 cell density reached 70% in 60 mm culture dishes, cells were incubated for 12 h with fresh RPMI 1640 medium (used as the control group), media containing PDA, AC, AP, or APM. Cells in the groups requiring NIR treatment were then exposed to 808-nm NIR irradiation (1.5 W/cm^2^, 10 min) and further incubated for 12 h. The cells were harvested and resuspended in radioimmunoprecipitation assay (RIPA) buffer (NCM Biotech, China) with phenylmethylsulfonyl fluoride (PMSF) (BOSTER, USA) to prevent protein degradation. Protein quantification was conducted using the BCA Protein Assay Kit (BOSTER, USA). Western blotting (WB) was performed to measure the expression levels of HMGB1, CRT, CD47, and COX-2. The level of immunosuppressive factor PGE2 in the supernatant of 4T1 cells was measured using an ELISA kit (Bioswamp, China).

### BMDC isolation and culture

Bone marrow-derived dendritic cells (BMDCs) were isolated from the femurs and tibias of 6- to 8-week-old male C57BL/6J mice. The cells were cultured in RPMI 1640 medium with 10% FBS and granulocyte-macrophage colony-stimulating factor (GM-CSF; 20 ng/ml, BioLegend) to promote BMDC maturation. After colony formation, BMDCs were cocultured with 4T1 cells and pretreated with different formulations for 24 h. BMDC was stained by anti-mouse CD11c-allophycocyanin (APC), CD80-fluorescein isothiocyanate (FITC), and CD86-phycoerythrin (PE) antibodies (BD Biosciences) and analyzed by FCM.

### Biodistribution and tumor-targeting capability

For in vivo biodistribution studies, 4 × 10^6^ 4T1 cells (100 μl) were injected into the mammary glands of female BALB/c mice. When the tumors reached approximately 100 mm^3^ on day 7, 4T1 tumor-bearing mice were injected via the tail vein with either free DiR or DiR-labeled nanoparticles (AP@DiR or APM@DiR). Fluorescence imaging of DiR signals was performed at 2, 12, and 24 h after injection using the IVIS Spectrum (PerkinElmer, USA). Ex vivo biodistribution was assessed in tumors and major organs (heart, liver, spleen, lung, and kidney).

### In vivo photothermal performance

For in vivo photothermal performance assessment, 4T1 tumor-bearing mice were administered with 100 μl of PBS, AP, or APM (AC: 2.5 mg/kg) via tail vein injection. Twenty-four hours later, infrared thermal images of the tumor site during NIR irradiation (1.5 W/cm^2^, 5 min) were captured at various time points using an infrared thermal camera.

### In vivo antitumor efficacy evaluation

An orthotopic breast cancer mouse model was established by injecting 4T1 cells into the mammary glands of female BALB/c mice. When the tumors reached approximately 100 mm^3^ on day 7 after inoculation, mice were randomly assigned to 7 groups (*n* = 5 per group) and administered with 100 μl of PBS, PDA, AC, AP, or APM (AC: 2.5 mg/kg) via tail vein injection every other day for a total of 3 doses. On the day following each injection, 3 treatment groups underwent NIR irradiation (1.5 W/cm^2^, 5 min). Body weight and tumor volume were monitored every other day. After 14 d, mice were euthanized, and tumors and major organs were collected for analysis. Tumor size and weight were measured, and tumors were fixed overnight in 4 % formaldehyde. Fixed tumors were paraffin-embedded and sectioned, followed by hematoxylin and eosin (H&E) staining and immunohistochemical Ki67 staining. The 4T1 breast cancer model is known for its high invasiveness and propensity to form spontaneous lung metastases [34–36]. The lung tissues from the breast tumor model were imaged and analyzed using H&E staining to evaluate spontaneous lung metastasis.

### In vivo antitumor immune effect evaluation

To evaluate the activation of antitumor immunity, tumors and lymph nodes from tumor-bearing mice were processed into single-cell suspensions. FCM was used to determine the proportions of immune cells, including M1 macrophages, mature DCs, and CTLs, using fluorescently labeled antibodies. These included BV510 Fixable Viability Stain 510, anti-CD16/32 monoclonal antibody, APC-Cy7 anti-CD45, FITC anti-CD3, APC anti-CD11c, BB515 anti-CD11b, BB700 anti-IA/IE, PE-Cy7 anti-CD86, PE anti-CD80, and PE-Cy7 anti-CD8 antibodies (BD Biosciences). Additionally, the level of factors PGE2 (Bioswamp, China) and interferon-γ (IFN-γ) (BioLegend, USA) in tumor tissues was measured using an ELISA kit.

### Biosafety assessment

To assess biocompatibility, fresh mouse whole blood was mixed with 0.9% NaCl and incubated with various formulations at 37 °C for 1 h. Negative and positive controls included 0.9% NaCl solution and deionized water, respectively. Hemolysis was evaluated by measuring the absorbance of the supernatant at 545 nm using an absorbance reader (INFINITE F50, TECAN, China). The body weight of the mice was recorded throughout the treatment period. Blood samples were collected to measure serum levels of glutamate aminotransferase (AST), alanine aminotransferase (ALT), urea (Urea), and creatinine (Cr). Major organs were fixed overnight in 4% formaldehyde and processed for H&E staining to evaluate tissue integrity and potential damage.

### Statistical analysis

Data are expressed as mean ± SD. Statistical significance was analyzed using GraphPad Prism 9.5.0 (GraphPad Software, San Diego, CA, USA). Comparisons between the 2 groups were made using a 2-tailed Student’s *t* test. For comparisons among multiple groups, one-way analysis of variance (ANOVA) followed by Tukey’s post hoc test was employed.

## Results and Discussion

### Preparation and characterization of APM

**Fig. 1.**
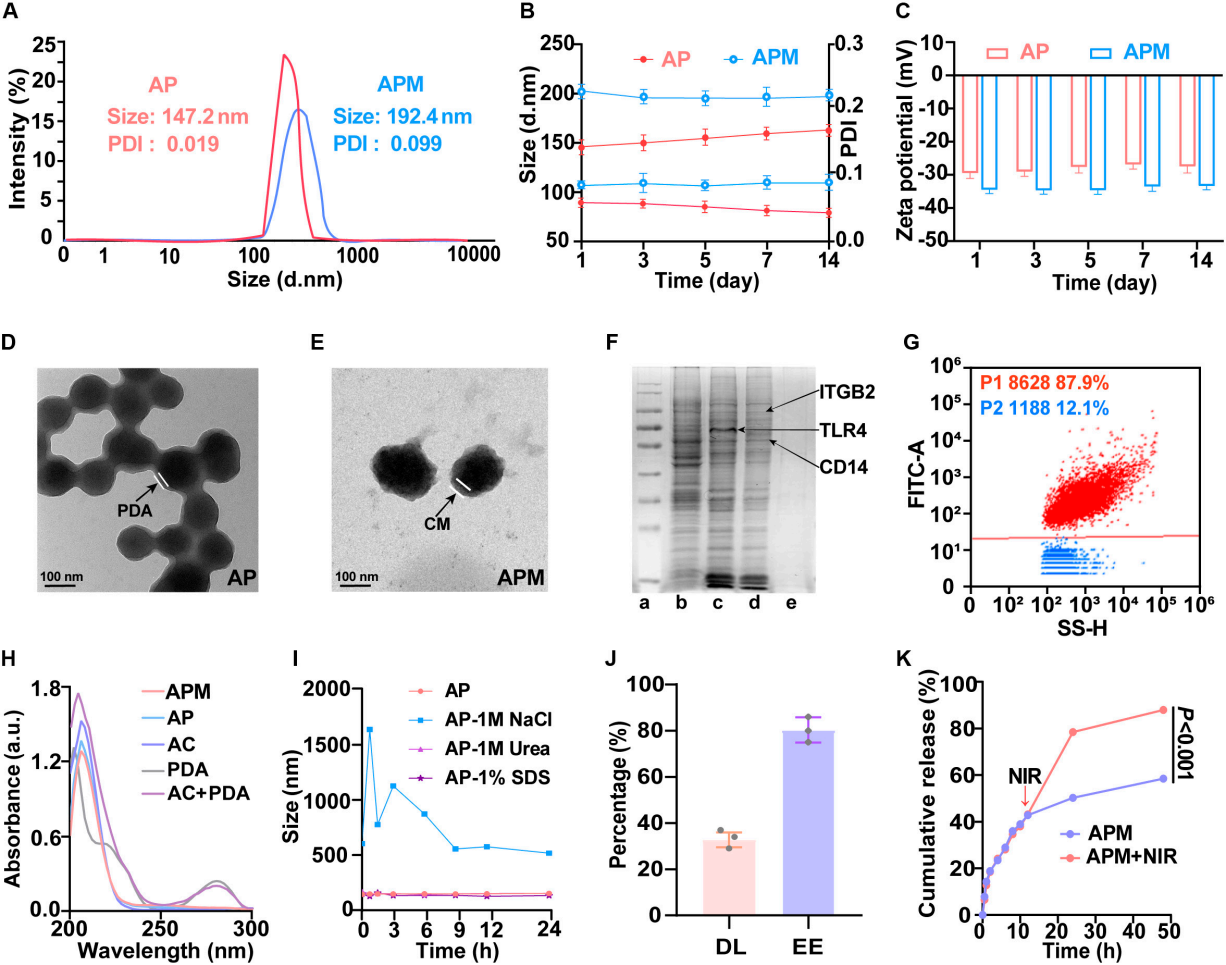
Characterization of AP and APM. (A) Particle size distribution and (B) stability analysis of AP and APM. (C) Zeta potential and stability analysis of AP and APM. (D) TEM micrographs of AP and (E) APM. (F) Coomassie brilliant blue analysis of (a) marker, (b) RAW 264.7 macrophage, (c) RAW 264.7 macrophage membrane (CM), (d) APM, and (e) AP. (G) Nano-flow cytometry analysis of CM encapsulation efficiency (P1: population of APM successfully encapsulated by the DiO-labeled CM, P2: population of unencapsulated AP). (H) UV–Vis absorption spectra of PDA, AC, AP, and APM, and (I) particle size change of AP in NaCl, Urea, and SDS solutions. (J) Drug loading and encapsulation efficiency of APM. (K) In vitro drug release profiles from APM and APM with NIR. Data are shown as mean ± SD (*n* = 3).

The hydrodynamic diameter of AP was 147.2 ± 17.5 nm (Fig. 1A). The coating of CM onto AP increased the diameter to 192.4 ± 30.6 nm (Fig. 1A). Besides, the zeta potential of AP shifted from −29.1 ± 0.2 mV to −35.8 ± 0.3 mV after CM coating, suggesting successful CM coting of AP to form APM (Fig. 1C). The increased negative zeta potential is likely due to the inherent properties of the CM, which is rich in negatively charged substances such as phospholipids and sialic acid residues [37]. These components dominate the nanoparticle surface after coating, which may enhance stability through improved electrostatic repulsion in biological environments. The PDI values of AP and APM were 0.019 and 0.099, respectively, indicating narrow particle size distributions, which are crucial for reproducibility and scalability (Fig. 1A). Stability tests showed no remarkable changes in hydrodynamic diameter, zeta potential, and PDI of APM in deionized water over 14 d (Fig. 1B and C). TEM images demonstrated that both AP and APM nanoparticles were spherical (Fig. 1D and E). The PDA layer appears as a uniform, continuous thin coating approximately 5 to 10 nm thick, exhibiting moderate electron density, consistent with PDA’s self-assembly properties (Fig. 1D). In contrast, the cell membrane (CM) layer presents a thicker coating, around 20 to 30 nm, with irregular electron density, likely due to the presence of the lipid bilayer and membrane proteins (Fig. 1E). Coomassie brilliant blue staining confirmed the retention of characteristic proteins from CM within APM, such as integrin β2 (ITGB2) and CD14 (Fig. 1F) [38,39]. Notably, the prominent band observed in lane C corresponds to Toll-like receptor 4 (TLR4) [40], which is highly expressed in CM (Fig. 1F). However, its minimal expression on APM may be due to the specific lipid environment and structural stability required for TLR4 [41], which may not be fully preserved during the transfer process. Despite this limitation, the successful incorporation of functional membrane proteins like CD14 and ITGB2 demonstrates the effectiveness of the CM encapsulation, thereby ensuring the biological functionality of APM for targeted therapeutic applications. Furthermore, nano-flow cytometry analysis indicated a high CM coating efficiency of 87.9% (Fig. 1G).

From the result of UV–Vis spectroscopy, AC exhibited a strong peak of around 210 nm, while PDA showed a peak of around 280 nm (Fig. 1H). The simple mixture of PDA and AC exhibited absorption peaks corresponding to each component (Fig. 1H), with no notable shift or new peak formation. In contrast, the AP complex exhibited an absorption spectrum similar to AC, lacking the characteristic PDA absorbance at 280 nm. This suggests that strong intermolecular interactions between PDA and AC suppress PDA’s typical absorbance. Notably, the absorption spectra of AC were varied in 1 M NaCl solution (Fig. S1). Besides, exposure to NaCl caused irregular changes to the particle size of AP, with PDI values exceeding 1 (Fig. 1I). This phenomenon can be attributed to the PDA coating binding predominantly to the AC drug core via electrostatic interactions, which rely on nanoparticle surface charges to maintain structural stability. In NaCl solution, the high concentration of Na^+^ and Cl^−^ ions neutralizes and shields the surface’s negative charges through ionic interactions. Consequently, the reduced electrostatic repulsion may cause partial detachment of the PDA coating or enhanced nanoparticle aggregation, compromising their stability. Conversely, no obvious alternations in the absorption spectrum or particle size were observed when AP was dispersed in 1 M urea or 1% SDS solutions (Fig. S1), suggesting that hydrogen bonding and hydrophobic interactions do not play a predominant role in the structural integrity of AP. Additionally, the spectra and size of APM showed minor changes across different solvents compared to AP (Figs. S2 and S3). This suggests that CM effectively shields the core and improves the stability of the nanoparticles.

The drug loading of APM was determined to be 37.8% ± 2.6%, with an encapsulation efficiency of 80.1% ± 3.0% (Fig. 1J). This high drug payload and efficient encapsulation are expected to augment therapeutic efficacy. Notably, NIR irradiation significantly accelerated the drug release from APM (*P* < 0.001), reaching a cumulative drug release of approximately 80% (Fig. 1K). Compared to traditional drug delivery systems such as micelles and liposomes [4,18], the membrane-coated self-assembled nanoparticles (APM) not only exhibit improved drug-loading capacity and stability but also leverage the photothermal properties of PDA. This enables enhanced and controlled drug release at tumor sites under NIR irradiation. In summary, these features are advantageous for enhancing therapeutic outcomes in cancer treatment.

### Photothermal performance of APM

**Fig. 2.**
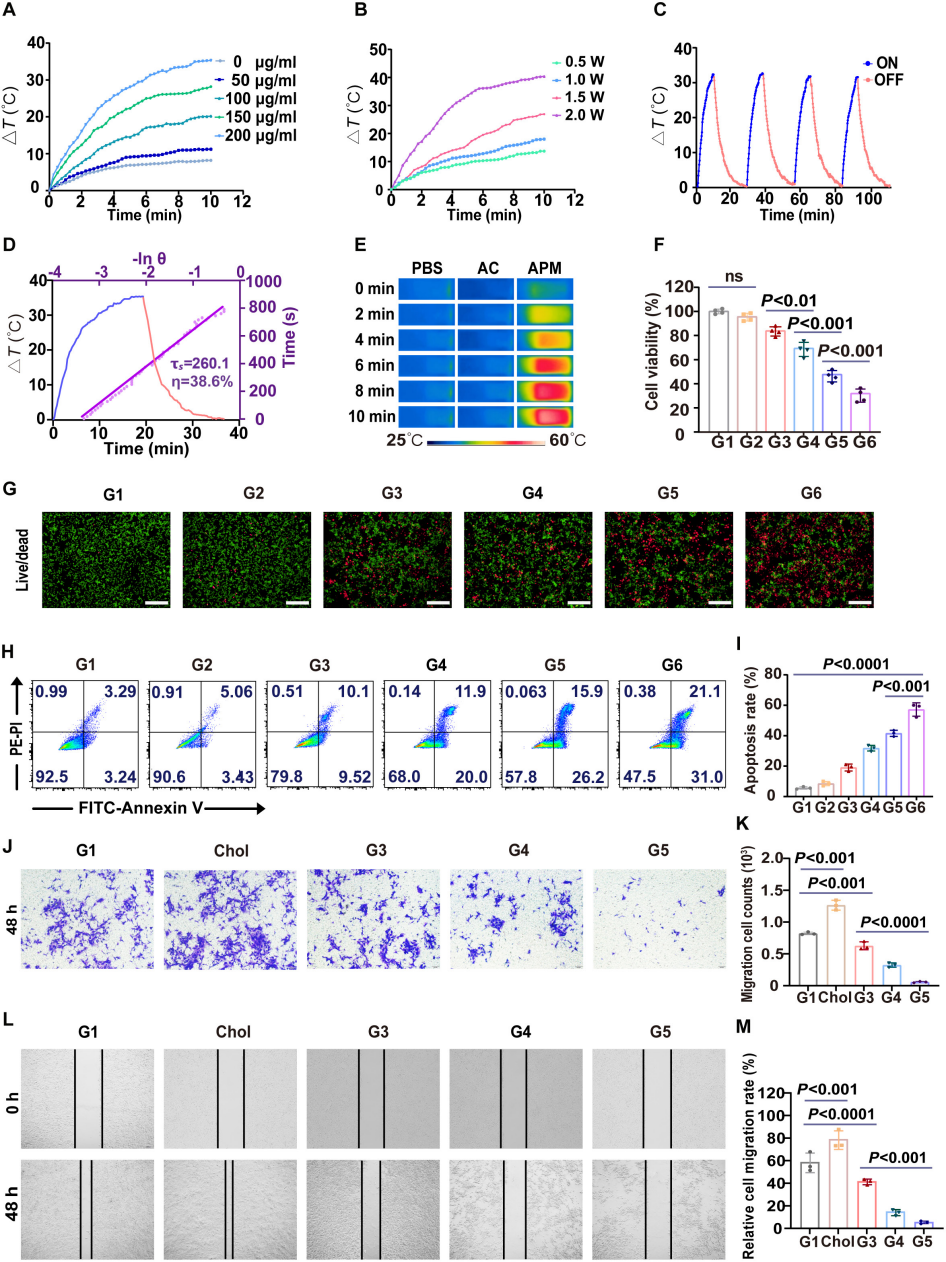
Photothermal performance and in vitro antitumor efficacy of APM. (A) Temperature change profiles of APM at various concentrations, (B) at various power densities of NIR, and (C) under 4 irradiation/cooling cycles. (D) Temperature–time curves related to photothermal conversion efficiency, along with the linear relationship curves (between time and −ln △θ) of APM under 1 W/cm^2^ 808-nm NIR. (E) Photothermal images of PBS, AC, and APM. (F) Cell viability with 4T1 cells with various treatments (with the AC concentration of 40 μg/ml). (G) Fluorescent images of live/dead staining of various treatments. Scale bar, 200 μm. (H) The apoptosis rate of 4T1 cells of various treatments was analyzed by FCM and (I) the quantitative evaluation. (J) 4T1 cell invasion rate of various treatments in scratch wound healing assay and (K) the quantitative analysis. (L) 4T1 cell migration counts of various treatments in transwell assay and (M) the quantitative analysis. Data are shown as mean ± SD (*n* = 3) (G1: control, G2: PDA + NIR, G3: AC, G4: AP, G5: APM, G6: APM + NIR, Chol: cholesterol).

We examined the photothermal efficiency of APM containing varying concentrations of PDA (0, 50, 100, 150, and 200 μg/ml) when exposed to NIR irradiation (1 W/cm^2^, 10 min) (Fig. S4). Our results showed that with increasing PDA concentration, the temperature rise rate of the APM solution becomes faster (Fig. S4). The maximum temperature change (Δ*T*) was observed at 200 μg/ml of PDA, reaching approximately 35.0 °C (Fig. S4). Further experiments assessed the impact of APM concentration on Δ*T* when exposed to NIR at 1 W/cm^2^ (Fig. 2A). The temperature increase correlated positively with the nanoparticle concentration, achieving a peak Δ*T* of about 35.4 °C at 200 μg/ml (Fig. 2A). Additionally, elevating the power density from 0.5 to 2.0 W/cm^2^ remarkably increased Δ*T* of 100 μg/ml APM (Fig. 2B). These results demonstrated the potential of APM under NIR to achieve a high temperature for thermal damage in the target tumor area. Besides, the photothermal performance of APM showed no marked change over 4 cycles of heating and cooling (Fig. 2C), demonstrating the stability of APM as a robust photothermal agent. To quantify the photothermal conversion efficiency of APM, Δ*T* was continuously monitored over time under 808-nm NIR irradiation (1 W/cm^2^), covering both the heating process until the equilibrium temperature was attained and the subsequent cooling process (Fig. 2D). The time constant of heat transfer from the system was determined to be τ*_s_* = 260.1 s for 808 nm, and the photothermal conversion efficiency (η) of APM was calculated to be about 38.6% (Fig. 2D), demonstrating that APM could be a potent candidate for PTT application. Furthermore, we compared the thermal responses of AC and APM. Furthermore, we compared the thermal responses of AC and APM dissolved in PBS (200 μg/ml). The photothermal images show that APM exhibits the highest increase in Δ*T* when exposed to NIR irradiation (Fig. 2E). These findings confirm the substantial role of PDA in the photothermal properties of APM.

### In vitro antitumor efficacy of APM

The cytotoxic effects of various formulations (PDA, AC, AP, and APM) on 4T1 cells were assessed through a CCK-8 assay. We found that free PDA-treated cells under NIR irradiation had minimal impact on cell viability, which was as high as about 93% when incubated with a high concentration (80 μg/ml) of PDA (Fig. S5). Conversely, treatments with AC, AP, and APM with or without NIR stimulation exhibited a dose-dependent cytotoxicity (Fig. S5). Notably, AP demonstrated higher cytotoxicity than free AC (40 μg/ml) (*P* < 0.01) (Fig. 2F), which may be attributed to the enhanced cellular uptake. Moreover, APM reduced the cell viability by approximately 36% compared to AP (*P* < 0.001) (Fig. 2F). This enhancement may be contributed by the modification of CM, further improving cellular internalization of APM and leading to a stronger cytotoxic. Furthermore, APM with NIR significantly reduced cell viability to about 35% (*P* < 0.001) (Fig. 2F). The therapeutic enhancement is likely due to the NIR-induced thermal damage and the accelerated release of AC from the APM. Calcein AM/PI live/dead staining showed distinct patterns of live (green fluorescence) and dead cells (red fluorescence) (S6). Increased red fluorescence was consistent with the trend observed in the CCK-8 assay for the same treatments (Fig. 2G). Additionally, cells treated with APM (with the AC concentration of 40 μg/ml) and NIR exhibited a significantly highest apoptosis rate, markedly greater than APM treated (*P* < 0.001) and the control group (*P* < 0.0001) (Fig. 2I). These findings underscore the potent synergistic antitumor effects of APM, in which the therapeutic efficacy of AC is notably augmented by the photothermal properties of PDA. This dual-functional approach offers a promising avenue for enhancing cancer therapy efficacy.

### In vitro anti-metastasis effect of APM

Previous studies have reported that cholesterol can facilitate tumor cell proliferation and metastasis [42]. In transwell assays, 4T1 cells cultured in a cholesterol-enriched medium displayed an increase of approximately 60% in invasion over 48 h, compared to the control group (*P* < 0.001) (Fig. 2J and K). Similarly, scratch assays indicated an accelerated healing rate of about 78.5% in the cholesterol group (Fig. 2L and M). Notably, AC (40 μg/ml), which is known for its cholesterol-lowering properties, resulted in a reduction in 4T1 cell invasion by approximately 24.3% (*P* < 0.001) (Fig. 2J and K) and decreased the scratch healing rate by about 28.7% (*P* < 0.001) compared to the control group (Fig. 2L and M). These findings indicate that the down-regulation of cholesterol levels by AC may play a crucial role in inhibiting the migration and invasion of 4T1 cells. Further analysis revealed that APM resulted in approximately a dramatic 85.2% reduction in 4T1 cell invasion compared to the AC-treated cells (*P* < 0.0001) (Fig. 2J and K) and lowered the healing rate to about 34.1% (*P* < 0.001) (Fig. 2L and M). The pronounced anti-metastatic effects of APM are likely attributable to its enhanced cellular uptake, which amplifies the cholesterol-lowering impact of AC. In summary, APM emerges as an innovative drug delivery system that potentially regulates tumor cholesterol levels to amplify its anti-metastatic capabilities.

### Cellular uptake and tumor targeting of APM

**Fig. 3.**
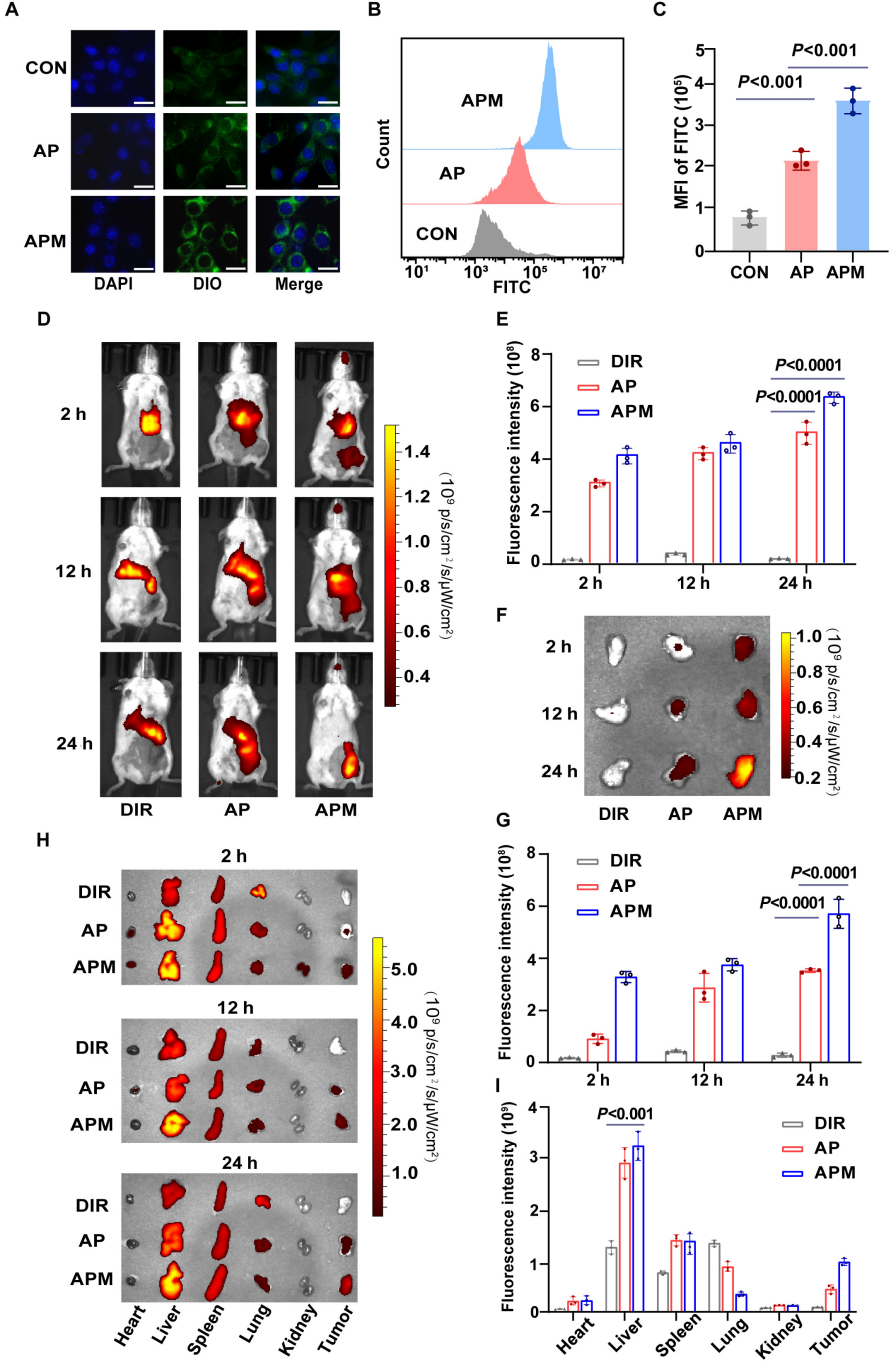
Cell uptake and biodistribution of AP and APM. (A) Fluorescence image of the 4T1 cell uptake. Scale bar, 20 μm. (B) 4T1 cell uptake was analyzed by FCM and (C) the quantitative analysis. (D) In vivo fluorescence images and (E) the quantitative analysis of mice at 2-, 12-, and 24-h points after injection of DiR, AP@DiR, and APM@DiR. (F) Ex vivo tumor imaging at 2, 12, and 24 h after injection in mice and (G) the quantitative analysis of DiR fluorescence in tumors. (H) Ex vivo major organ imaging at 2, 12, and 24 h after injection in mice and (I) the quantitative analysis of DiR fluorescence in organs at 24 h after injection in mice (CON: control).

The effective internalization of APM@DiO in 4T1 tumor cells was evidenced by strong green fluorescence (Fig. 3A), and the mean fluorescence intensity (MFI) of APM@DiO was about 1.8 times higher than AP@DiO treatment from the FCM analysis (*P* < 0.001) (Fig. 3B and C). These findings demonstrated that CM modification further promotes the tumor cell uptake efficiency of APM. Further, the biodistribution results demonstrated a progressive increase in fluorescence intensity within tumor tissues over time for both AP@DiR and APM@DiR (Fig. 3D to H). After 24 h, APM@DiR showed more accumulation in tumor regions than both AP@DiR (*P* < 0.0001) and DiR (*P* < 0.0001) (Fig. 3D and E), demonstrating the tumor-targeting capability of APM. Further analysis revealed that APM@DiR displayed approximately 1.6 times higher fluorescence intensity than AP@DiR (*P* < 0.0001) (Fig. 3F and G). The enhanced tumor-targeting capability of APM@DiR can be primarily attributed to the unique lipid and protein composition of the CM coating [43]. The lipid bilayer of the CM provides structural stability and functional specificity, while membrane proteins enable APMs to mimic macrophage interactions within the TME, which facilitates selective accumulation in tumor tissues [44]. Additionally, during the CM extraction process, residual organelle membranes, such as mitochondrial and endoplasmic reticulum (ER) membranes, may introduce heterogeneity. However, these organelle membranes possess intrinsic properties that can enhance intracellular targeting [45]. For instance, mitochondrial membranes may promote mitochondrial targeting, leading to increased ROS production, which triggers oxidative stress and amplifies ICD to augment antitumor effects [46]. Besides, APM@DiR exhibited the highest distribution in the liver (*P* < 0.001), likely due to its biomimetic properties, which help slow down metabolism and prolong retention time in vivo. Moreover, the persistent fluorescence signal in the head region of the APM group is likely due to the CM coating, which enhances interactions with the blood–brain barrier (BBB) via surface proteins such as integrins and adhesion molecules [47,48]**.** CM can also provide immune evasion, prolonging circulation time and promoting accumulation in the head region [49]. In contrast, DiR and AP lack these properties, resulting in rapid clearance and minimal fluorescence. These characteristics collectively underscore the advantages of CM-coated nanoparticles in enhancing tumor targeting and therapeutic efficacy.

### Mechanisms of APM in counteracting inflammation and tumor immunosuppression

**Fig. 4.**
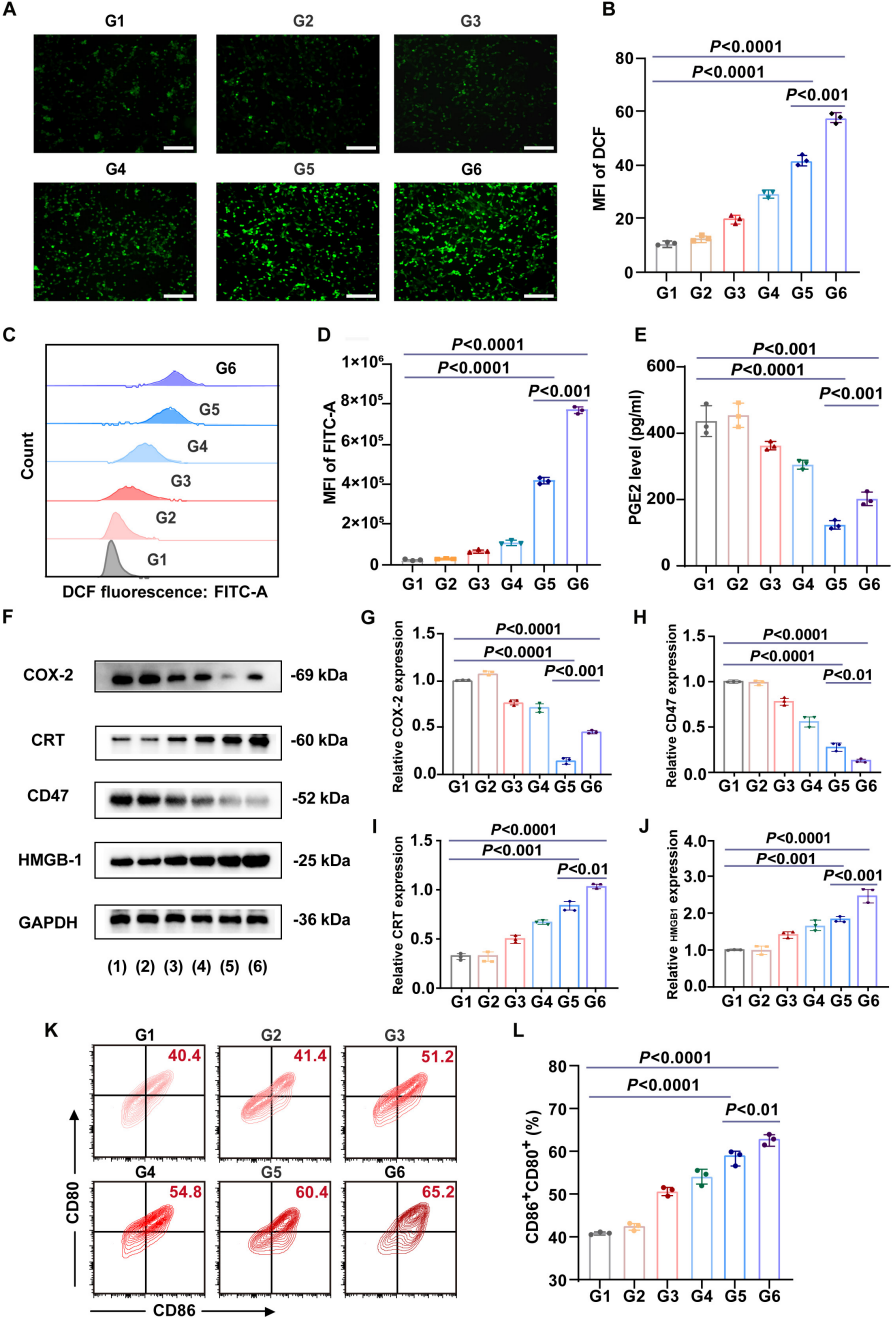
In vitro anti-inflammatory and antitumor immune activation effect of APM. (A) Fluorescence images of ROS in 4T1 cells under various treatments and (B) the quantitative assessment. Scale bar, 200 μm. (C) The expression levels of ROS in 4T1 cells were analyzed by FCM under various treatments and (D) quantitative assessment. (E) The expression levels of PGE2 in 4T1 cells were analyzed by ELISA under various treatments. (F) WB analysis of the expression trends of COX-2, CD47, CRT, and HMGB1 proteins in 4T1 cells under various treatments and (G to J) the quantitative assessment. GAPDH is shown as a representative loading control. Complete results are presented in Fig. S7. (K) DC maturation rate analyzed by FCM after incubation with 4T1 cells under various treatments and (L) the quantitative assessment. Data are shown as mean ± SD (*n* = 3) (G1: control, G2: PDA + NIR, G3: AC, G4: AP, G5: APM, G6: APM + NIR).

WB analysis revealed that APM treatment led to a significant decrease in COX-2 protein levels in 4T1 cells compared to the control group (*P* < 0.0001) (Fig. 4F and G). ELISA assays showed a reduction of about 72.1% in PGE2 concentrations in the supernatants of APM-treated cells compared to controls (Fig. 4E). When cells were treated with APM + NIR irradiation, there was an up-regulation in the expression levels of COX-2 and PGE2 compared to APM treatment (*P* < 0.001, *P* < 0.001) (Fig. 4G and E). This up-regulation may be associated with the PTT responses triggered by NIR in cells. Despite this, the combined treatment decreased levels of COX-2 and PGE2 compared to the controls (*P* < 0.0001, *P* < 0.0001) (Fig. 4E and G). This suggests that APM effectively inhibits the COX-2/PGE2 pathway, which could be potentially helpful for counteracting the drawback of PTT. Moreover, 4T1 cells treated with APM exhibited a significant reduction in CD47 expression compared to the control group (*P* < 0.0001) (Fig. 4F and H). When APM treatment was combined with NIR, CD47 expression was significantly higher than with APM treatment alone (*P* < 0.01) and control group (*P* < 0.0001) (Fig. 4F and H). These results suggest that the APM treatment with NIR could be helpful for inhibiting tumor immune evasion and enhancing the phagocytosis of macrophages against tumors.

### In vitro ROS generation and ICD by APM

Incubation of APM with 4T1 cells resulted in a notable increase in ROS production, evidenced by about a 2-fold increase in fluorescence intensity compared to the control (*P* < 0.0001) (Fig. 4A and B). FCM analysis further demonstrated these findings, showing significantly heightened ROS fluorescence in the APM-treated group compared to controls (*P* < 0.0001) (Fig. 4C and D). These results suggest that APM effectively stimulates ROS generation. Notably, when 4T1 cells were subjected to NIR irradiation in combination with APM, ROS production was further amplified (*P* < 0.001) (Fig. 4A to D), demonstrating that NIR irradiation potentiated the ROS-generating capacity of APM. Moreover, WB analysis revealed that APM treatment enhanced the expression of CRT and HMGB1 proteins in 4T1 cells (Fig. 4F). Specifically, CRT expression was approximately 2.5 times that of the control (*P* < 0.001), and HMGB1 expression was about 1.8 times that of the control (*P* < 0.001), as determined by semiquantitative ImageJ analysis of the bands (Fig. 4I and J). Notably, the APM + NIR group further elevated the expression of CRT and HMGB1 compared to the APM group (*P* < 0.01 and *P* < 0.001, respectively) (Fig. 4I and J), indicating that PTT may intensify the ICD process induced by APM.

The maturation of DCs is a pivotal step for eliciting robust immune responses. FCM analysis revealed that APM-treated 4T1 tumor cells stimulated a substantial increase in the expression of key maturation markers (CD86 and CD80) on BMDCs. Specifically, there was approximately a 2-fold increase in APM-treated BMDCs compared to the untreated control (*P* < 0.0001) (Fig. 4K and L). Furthermore, the addition of NIR irradiation markedly enhanced the maturation of BMDCs compared to the treatment with APM alone (*P* < 0.01) (Fig. 4K and L), demonstrating that APM with NIR irradiation can promote DC activation stimulated by tumor cell death. This effect underscores the potential of APM and PTT to reinforce the antitumor immune response.

### In vivo antitumor efficiency of APM

**Fig. 5.**
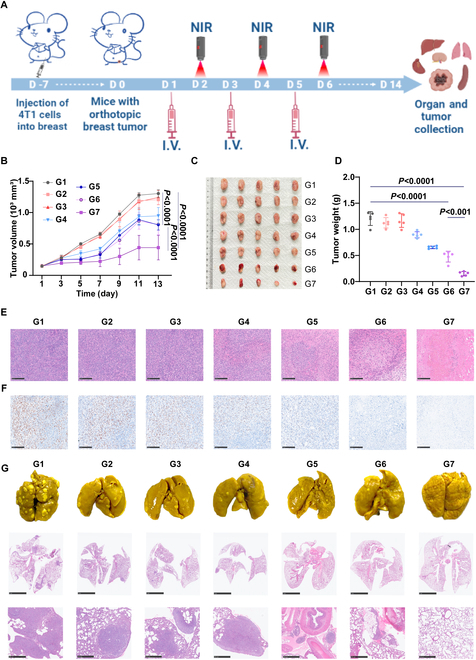
In vivo antitumor efficacy of APM. (A) Schedule of various treatments in orthotopic tumor-bearing mice. (B) Tumor volume change curves in orthotopic tumor-bearing mice under various treatments during 14 d of treatment and observation. (C) Ex tumors images and (D) tumor weight in orthotopic tumor-bearing mice under various treatments on the 14th day. (E) H&E staining and (F) Ki67 staining images of tumors in orthotopic tumor-bearing mice under various treatments. Scale bar, 250 μm. (G) Representative images of lung metastases from each treatment group. The top row shows the gross morphology of lung surfaces, where white nodules indicate metastatic lesions. The middle and bottom rows display H&E-stained lung sections at low (scale bar, 5 mm) and high (scale bar, 500 μm) magnifications. Dense, deeply stained nodules highlight metastatic foci within the lung parenchyma. Quantitative data on the area of metastatic nodules are provided in Fig. S11. Data are shown as mean ± SD (*n* = 5) (G1: control, G2: PDA + NIR, G3: AC, G4: AP, G5: AP + NIR, G6: APM, G7: APM + NIR, NIR laser: 808 nm, 1.5 W/cm^2^).

We evaluated the in vivo antitumor efficacy of APM using a murine orthotopic breast cancer model (Fig. 5A). Following the administration of APM, the temperature at the tumor site was measured to evaluate the photothermal effects under NIR radiation. Twenty-four hours after administration, the tumor site temperature remarkably increased to about 60 °C (Fig. S8) when subjected to NIR radiation (1.5 W/cm^2^, 5 min). The temperature increase was substantially higher than the PBS control group (about 38 °C) (Fig. S8). Subsequently, tumor-bearing mice with tumor volumes of approximately 100 mm^3^ were randomly divided into 7 treatment groups (*n* = 5): PDA + NIR, AC, AP, AP + NIR, APM, APM + NIR, and PBS control. Over 14 d, rapid tumor growth was observed in the PBS, PDA + NIR, and AC groups (Figs. S9 and S10), with tumors reaching an average volume of about 1,300 mm^3^ (Fig. 5B and C). In contrast, the AP-treated group exhibited slower tumor growth (Figs. S9 and S10), reducing tumor volumes to an average of 850 mm^3^ (*P* < 0.0001) (Fig. 5B and C). The AP + NIR and APM groups displayed moderate tumor suppression (Figs. S9 and S10), reducing average tumor volumes to approximately 650 mm^3^ (*P* < 0.0001) (Fig. 5B and C). Notably, the APM + NIR treatment achieved significant tumor suppression (Figs. S9 and S10), resulting in an average tumor volume of about 200 mm^3^ (*P* < 0.0001) (Fig. 5B and C). Besides, the tumor weight change revealed a tumor growth inhibition (TGI) rate of approximately 41.7% in the APM group, which was augmented to about 75.0% in the APM + NIR group (*P* < 0.001) (Fig. 5D). These results highlight the synergistic effects of APM formulation combined with NIR-triggered PTT, which exhibited the most pronounced antitumor efficacy among all treatment groups.

H&E staining showed extensive apoptosis and necrotic cells in the tumor tissues of mice treated with APM + NIR (Fig. 5E). Conversely, tissues from other treatment groups exhibited varying degrees of tumor cell death, with some cells still showing condensed chromatin and partially preserved nuclear morphology. The difference in H&E staining highlights that APM + NIR treatment holds a potent antitumor effect compared to the other treatments. Additionally, the APM + NIR group demonstrated the lowest level of Ki67 (Fig. 5F), indicating the most effective inhibition of tumor proliferation. Furthermore, the APM + NIR group displayed no presence of lung metastasis, while other groups showed different degrees of metastasis in lung tissue (Fig. 5G). The number of metastatic nodules was significantly reduced in the APM + NIR group compared to controls (*P* < 0.001) (Fig. S11), indicating the efficacy of the combined treatment in inhibiting lung metastasis. Overall, APM combined with NIR not only effectively suppressed tumor growth but also prevented tumor metastasis, providing a robust strategy for cancer therapy.

### In vivo anti-inflammatory and antitumor immune responses of APM

**Fig. 6.**
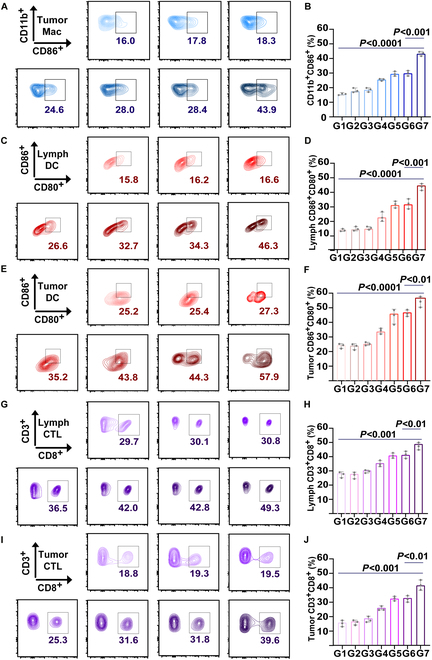
In vivo antitumor immune activation assay of APM. (A) M1 macrophage rate analyzed by FCM in tumors under various treatments and (B) the quantitative assessment. (C) DC maturation rate analyzed by FCM and (D) the quantitative assessment in tumors and (E and F) lymph nodes under various treatments. (G) CTLs rate analyzed by FCM and (H) the quantitative assessment in tumors and (I and J) lymph nodes under various treatments. Data are shown as mean ± SD (*n* = 3) (various treatments including G1: control, G2: PDA + NIR, G3: AC, G4: AP, G5: AP + NIR, G6: APM, G7: APM + NIR; the details of the gating strategy for all FCM data are in Fig. S13).

To explore the anti-inflammatory properties of APM in vivo, we assessed PGE2 levels in tumor tissues following various treatments (Fig. S12). PGE2 levels in the APM + NIR group showed an increase of approximately 20.3% compared to the APM group (*P* < 0.001), yet these levels were substantially lower than those observed in the PBS group (*P* < 0.0001) (Fig. S12). This suggests that APM effectively inhibited the inflammatory effects induced by PTT, which is beneficial to reducing immunosuppression within TME. Further immunological profiling was conducted using FCM to analyze immune cell subsets in the treated mice. Specifically, the proportion of M1 macrophages (CD11b^+^CD86^+^) in tumors of the APM + NIR group increased by about 15.5% (*P* < 0.001) and 27.9% (*P* < 0.0001) compared to the APM and PBS groups, respectively (Fig. 6A and B and Fig. S13). Besides, compared to the APM group, the maturation of DCs (CD80^+^CD86^+^) in the APM + NIR group increased by about 12.0% in lymph nodes (*P* < 0.001) and 13.6% in tumor (*P* < 0.01) (Fig. 6C to F and Fig. S13). Moreover, when compared to the PBS group, the maturation of DC in the APM + NIR group approximately increased by 30.5% in lymph nodes (*P* < 0.0001) and 32.6% in tumor (*P* < 0.0001) (Fig. 6C to F and Fig. S13), demonstrating a significant improvement of DC activation under the synergistic treatment. In the APM + NIR group, CTLs (CD3^+^CD8^+^) increased by about 6.5% (in lymph nodes, *P* < 0.01) and 7.8% (in tumor, *P* < 0.01) compared to the APM group, and by about 19.6% (in lymph nodes, *P* < 0.001) and 20.8% (in tumor, *P* < 0.001) compared to the PBS group (Fig. 6G to J and Fig. S13). These findings demonstrate an augmentation in the infiltration of CTLs within both lymph nodes and tumor tissues. Besides, the ELISA results showed that APM + NIR treatment significantly increased IFN-γ levels compared to all other groups, with the highest levels observed in the APM + NIR group (*P* < 0.0001 versus control, *P* < 0.001 versus APM) (Fig. S14). These findings are consistent with the FCM data, which demonstrated a marked increase in tumor-infiltrating CD3^+^CD8^+^ CTLs (Fig. 1I and J). Together, these data highlight the ability of APM + NIR to induce ICD, activate APCs, and stimulate CTLs, resulting in robust antitumor immune responses. This provides robust experimental evidence for photothermal synergistic cancer immunotherapy of APM.

### Biosafety assessment

The biosafety of our formulations was evaluated starting with their hemolytic activity, which showed no marked hemolytic phenomena compared to the negative and positive control group (Fig. S15). During the 14-d study period, no notable changes in body weight were observed among the experimental groups (Fig. S16). Following euthanasia on day 14, blood samples and major organs were collected for further analysis. Biochemical blood analysis revealed that hepatic function indicators, such as ALT and AST, as well as renal function indicators, such as Urea and Crea, remained within the normal range for all groups, suggesting no systemic toxicity (Fig. S17). Histopathological analysis of the major organs (heart, liver, spleen, lung, and kidney) using H&E staining showed no evidence of marked organ damage (Fig. S18). In summary, the nanoparticle formulation demonstrated good biocompatibility and low toxicity. Nevertheless, while these preliminary findings are encouraging, extended studies are essential to thoroughly assess long-term toxic effects.

## Conclusion

In summary, we have developed a biomimetic drug delivery system APM with high drug-loading and efficient tumor-targeting capabilities. Moreover, leveraging the PTT effect of PDA, APM further augments the antitumor effects and antitumor immune responses of AC. Furthermore, the local inflammation-induced immunosuppressive TME from PTT was ameliorated by inhibiting the COX-2/PGE2 pathway and CD47 signaling by AC. These findings demonstrated that APM could provide a feasible strategy to enhance the antitumor efficacy of photothermal synergetic immunotherapy. In conclusion, this study presents a multifunctional AC and PDA-based nanomedicine for tumor therapy. Future efforts will concentrate on overcoming technical challenges, including ensuring long-term safety, enabling large-scale production, and developing personalized treatments. This approach aims to better cater to the diverse needs of cancer patients and facilitate the clinical translation of therapies.

## Ethical Approval

Animal experiments were performed according to the protocols approved by the Institutional Animal Care and Use Committees of the Department of Laboratory Animals of Second Xiangya Hospital, Central South University (no. 20241024).

## Data Availability

The data used in this study are available from the authors upon reasonable request.
