# Supplementary material for: Biomimetic Atorvastatin Self-Assembled Nanomedicine Inhibits the Cyclooxygenase-2/Prostaglandin E2 Pathway Enhanced Photothermal and Antitumor Immunity
Source: Biomater Res. 2025 Mar 4;29:0149. doi: 10.34133/bmr.0149 (PMC11876541; doi:10.34133/bmr.0149)
Supplement: Supplementary 1 — Figs. S1 to S18 [file bmr.0149.f1.docx]

**Biomimetic Atorvastatin Self-Assembled Nanomedicine Inhibit the Cyclooxygenase-2/prostaglandin E2 Pathway Enhanced Photothermal and Antitumor Immunity**

Min Zhou^1,2,3^, Ruyue Han^1,2,3^, Wenjie Xu^1,2,3^, Xinyan Hao^1,2,3^, Yanjin Peng ^1,2,3^, Yucheng Tang^1,2,3^, Pengcheng Sun^1,2,3^, Tiantian Tang^1,2,3^, Junyong Wu^1,2,3,4*^, Daxiong Xiang^1,2,3*^

^1^ Department of Pharmacy, The Second Xiangya Hospital, Central South University, 410011, Changsha, China

^2^ Hunan Provincial Engineering Research Centre of Translational Medicine and Innovative Drug, 410011, Changsha, China

^3^ Institute of Clinical Pharmacy, Central South University, 410011, Changsha, China

^4^ Hunan Key Laboratory of Tumor Models and Individualized Medicine, The Second Xiangya Hospital, Central South University, 410011, Changsha, China

*Correspondence: wujunyong@csu.edu.cn (J. Wu); xiangdaxiong@csu.edu.cn (D.

Xiang)

**
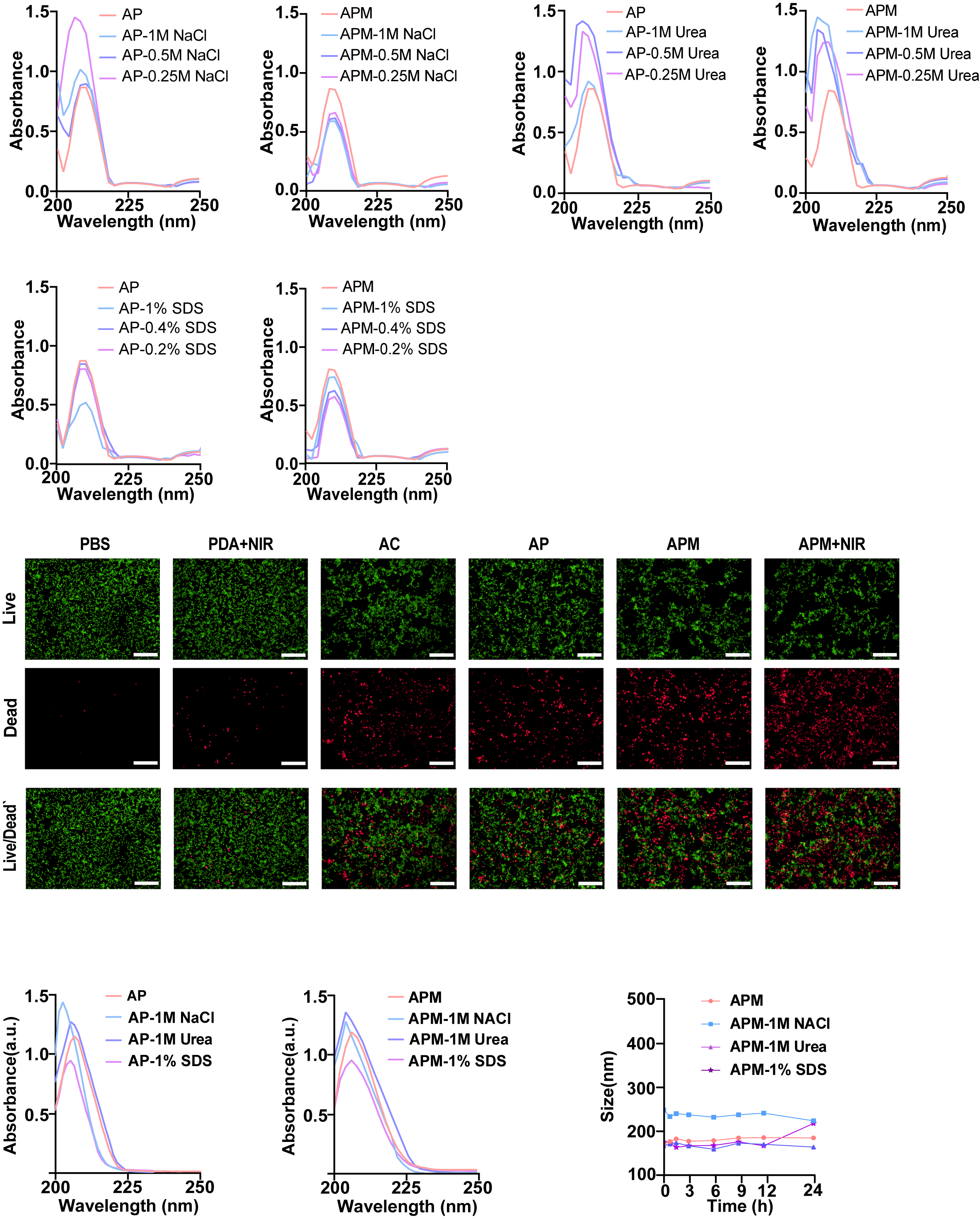
Supplementary results**


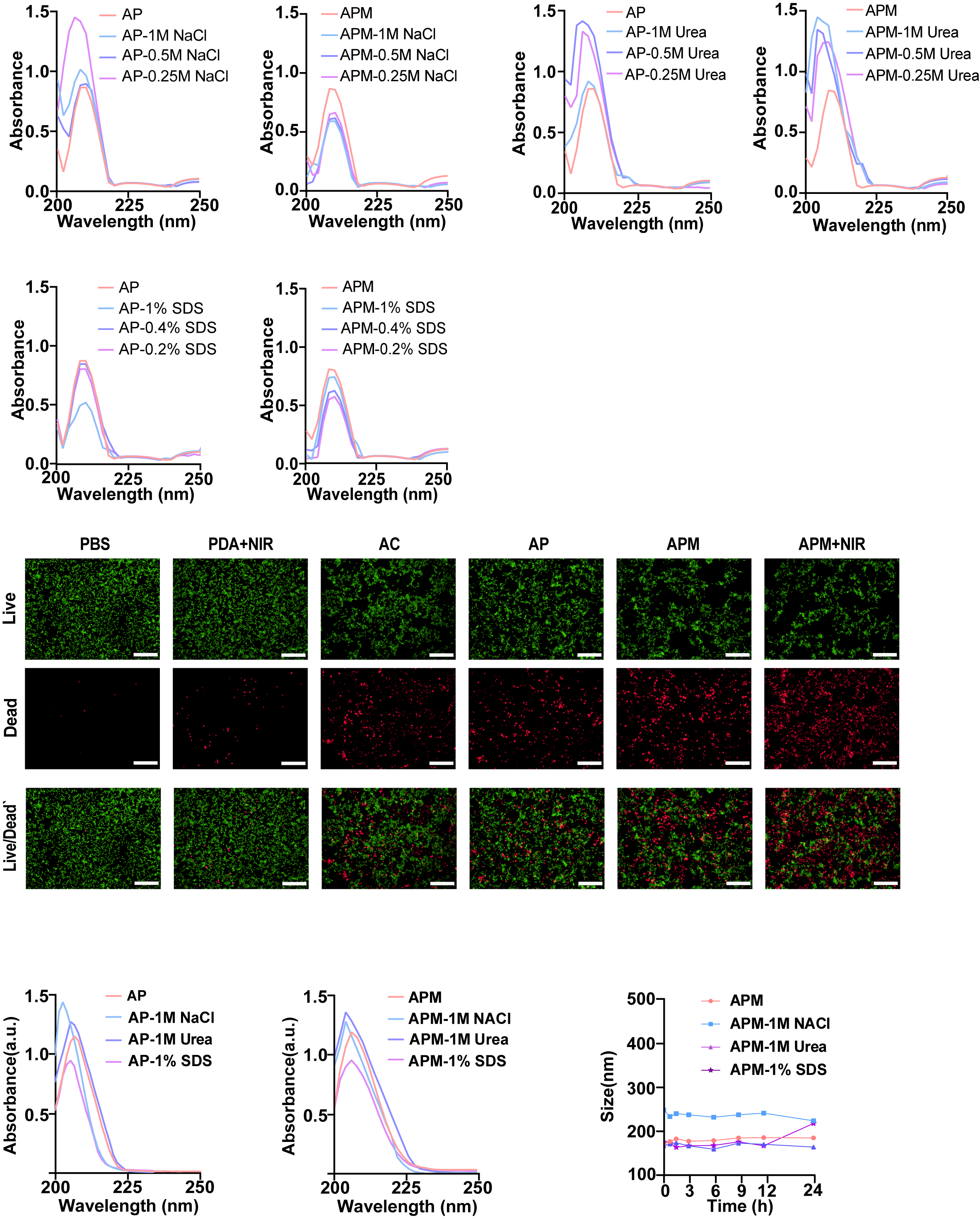
Figure S1. UV-Vis absorption spectra of AP in NaCl, Urea, and SDS solutions.

Figure S2. UV-Vis absorption spectra of APM in NaCl, Urea, and SDS solutions.


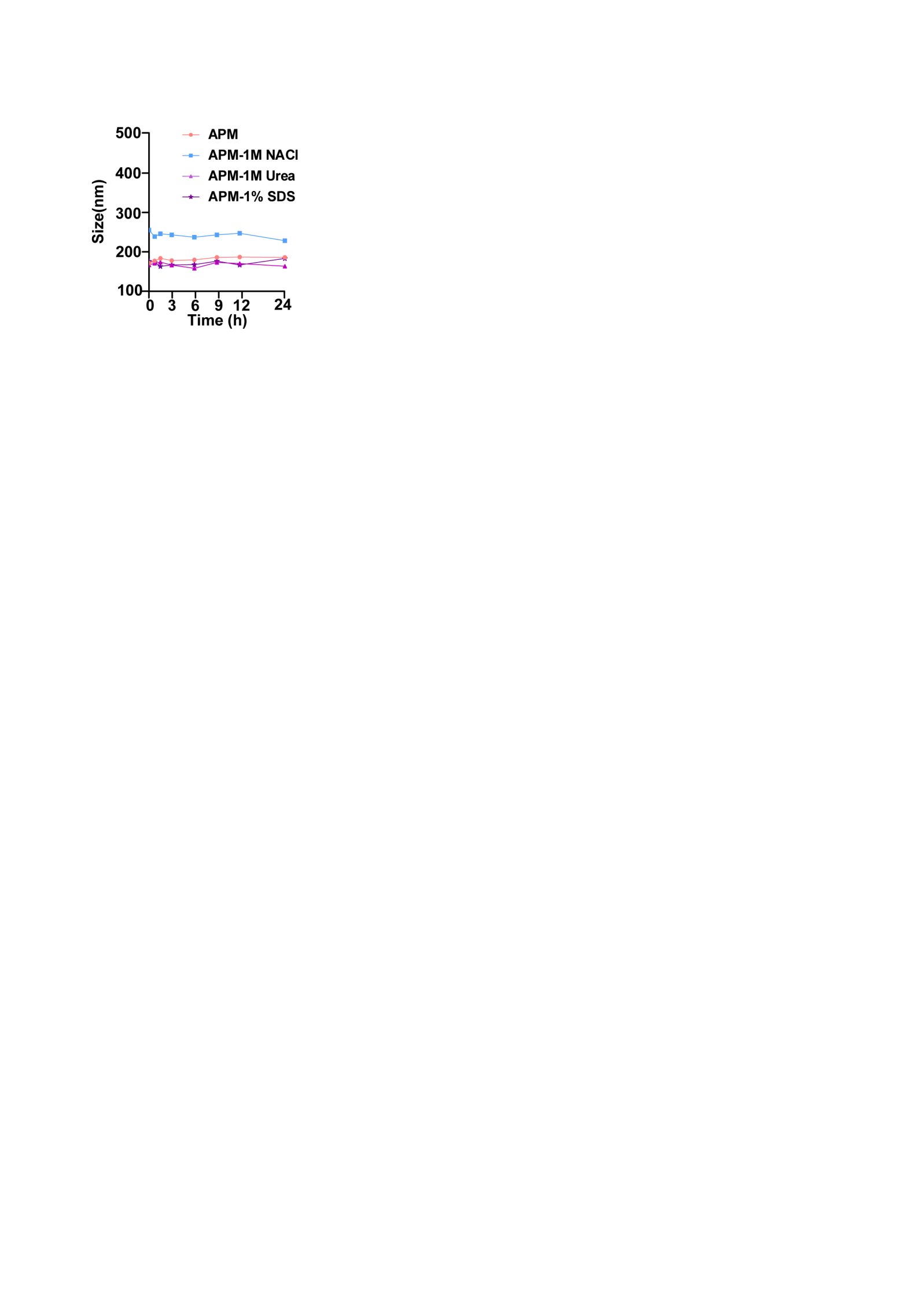


Figure S3. The particle size change of APM in NaCl, Urea, and SDS solutions.


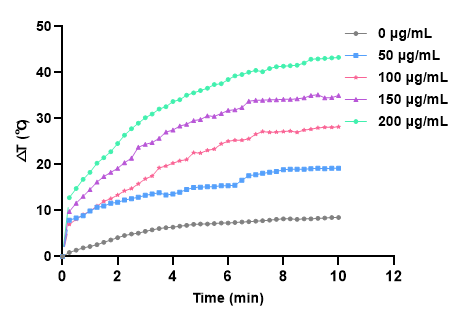


Figure S4. Temperature change profiles of APM at various concentrations of PDA.


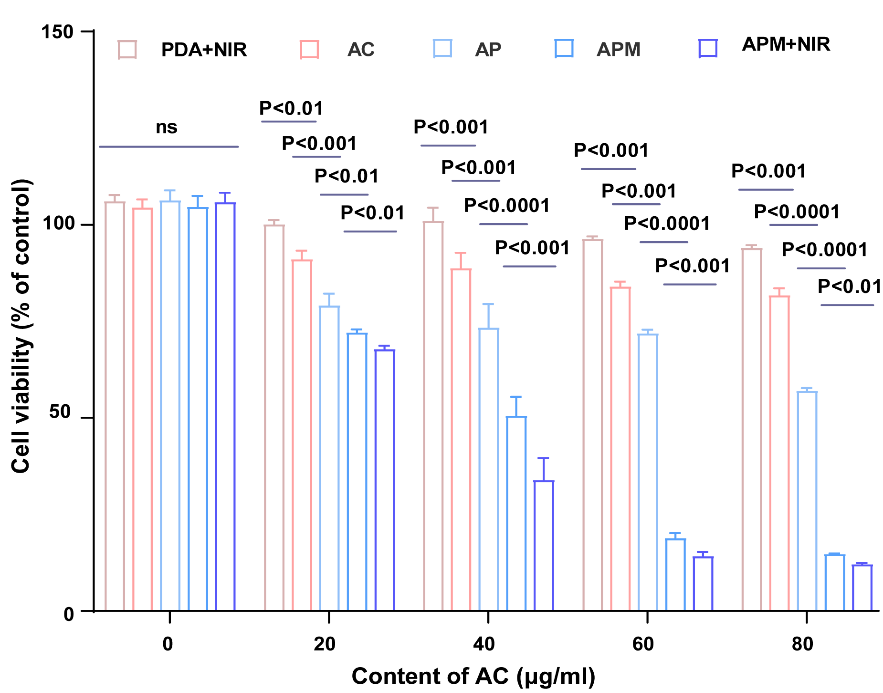


Figure S5. Cell viability with 4T1 cells with various treatments. (n=6)


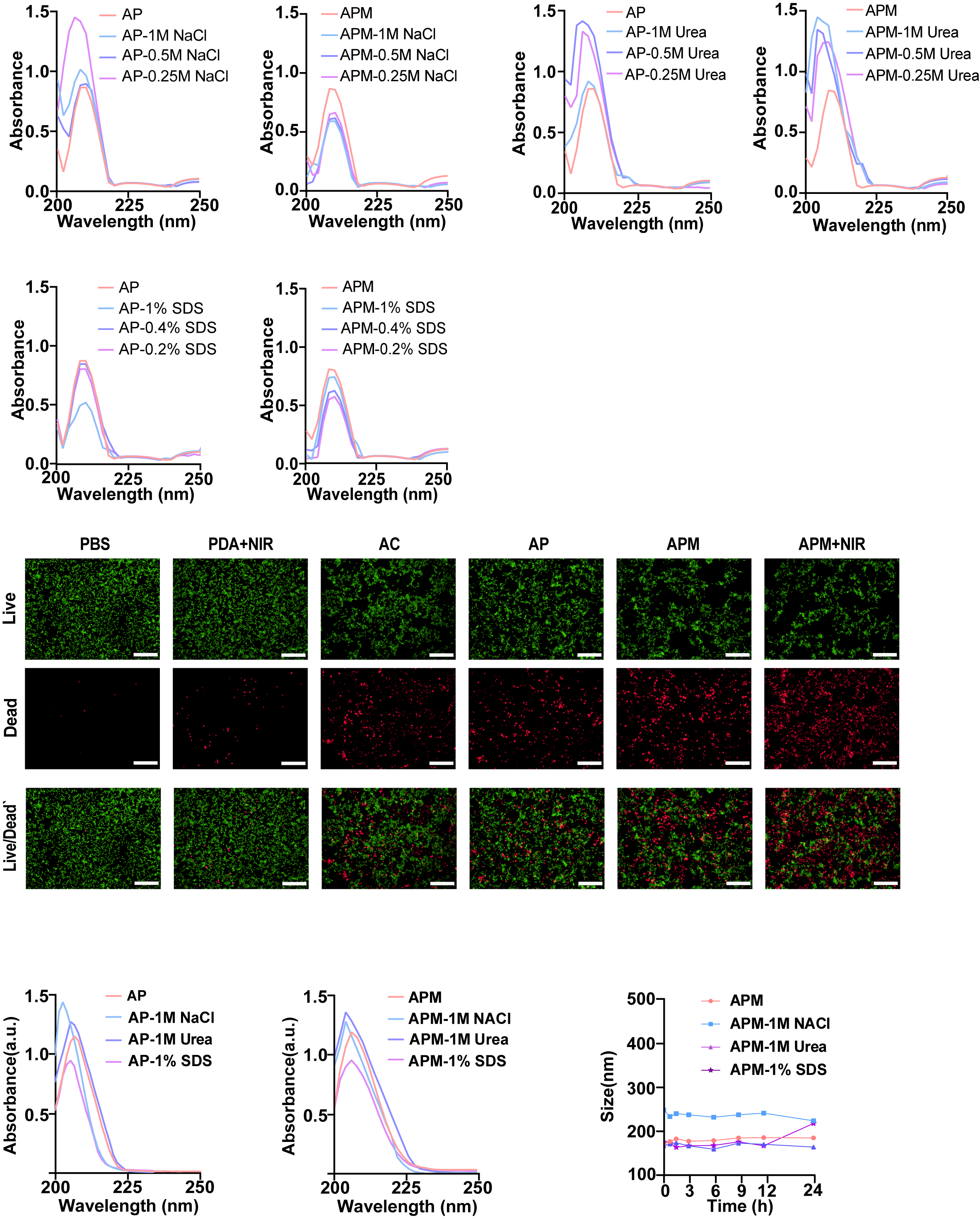


Figure S6. Fluorescent images of live/dead staining of various treatments. Scale bar =

200 μm.


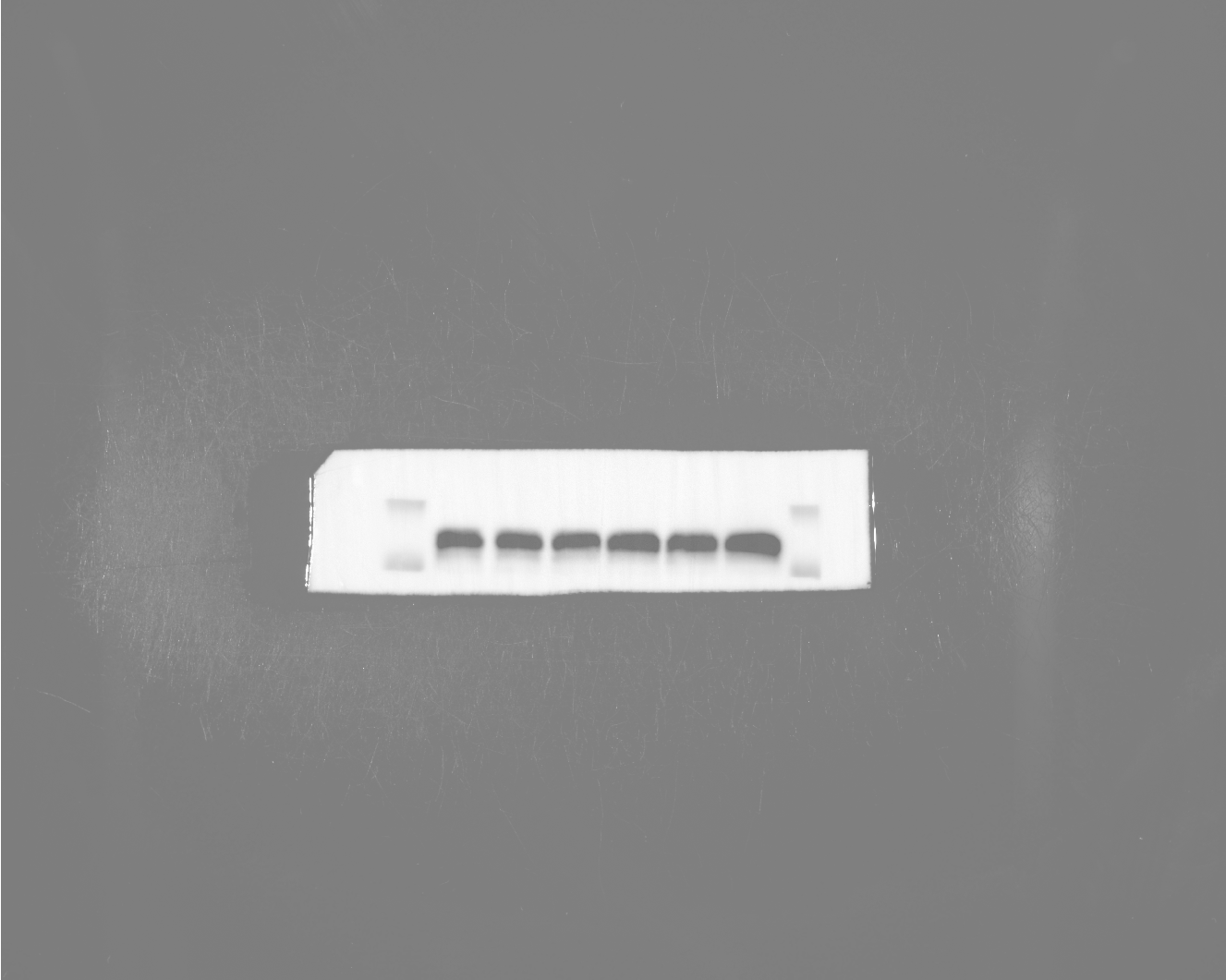

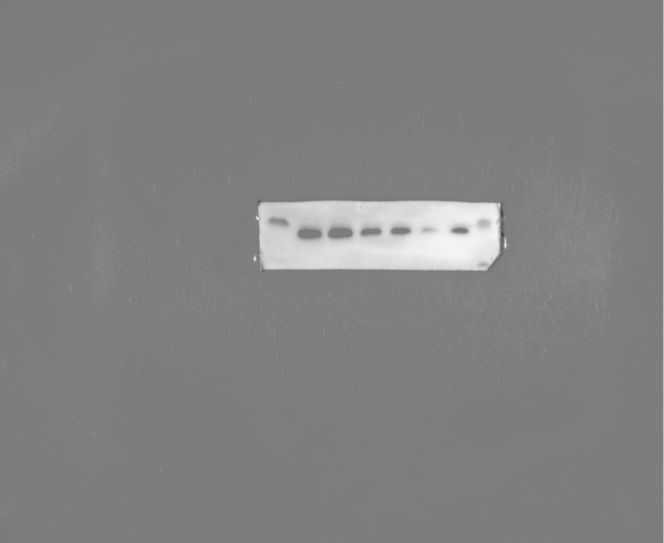

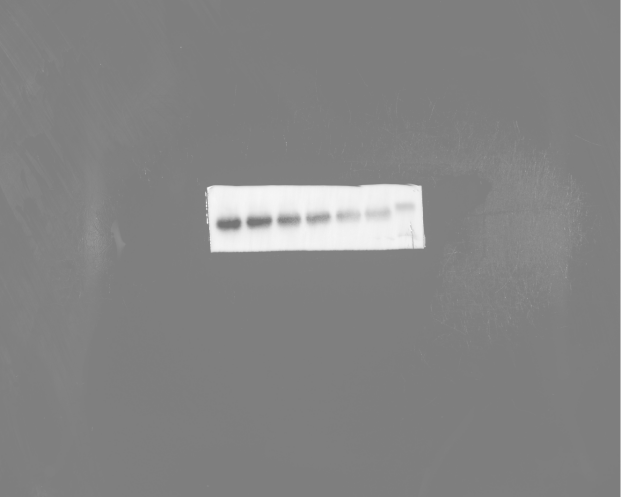

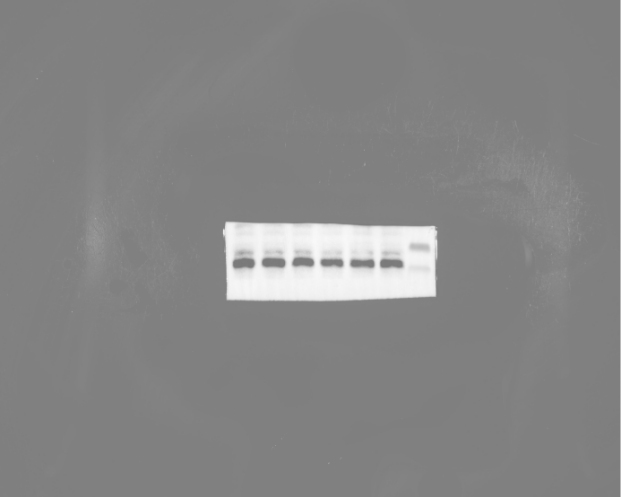

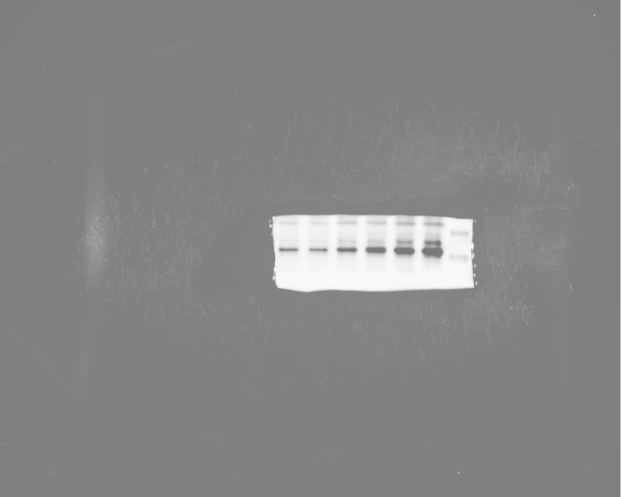

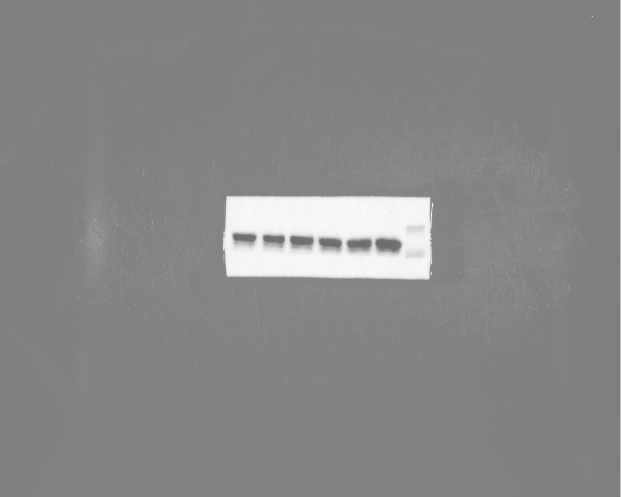


**A**

**B**

**CRT (60 kDa)**

**CD47 (52 kDa)**

**GAPDH (36 kDa)**

**HMGB1 (25 kDa)**

**COX-2 (69 kDa)**

**GAPDH (36 kDa)**

**70 kDa**

**55 kDa**

**55 kDa**

**40kDa**

**40 kDa**

**35 kDa**

**35 kDa**

**25 kDa**

**70 kDa**

**55 kDa**

**40 kDa**

**35 kDa**

Figure S7. Western blot analysis of protein expression in 4T1 cells under various treatments. (A) Protein expression levels of CRT (60 kDa), CD47 (52 kDa), GAPDH (36 kDa), and HMGB1 (25 kDa) on one membrane. (B) Protein expression levels of COX-2 (69 kDa) and GAPDH (36 kDa) on another membrane. GAPDH was employed as a loading control. Molecular weight markers are indicated on the right side of each panel.


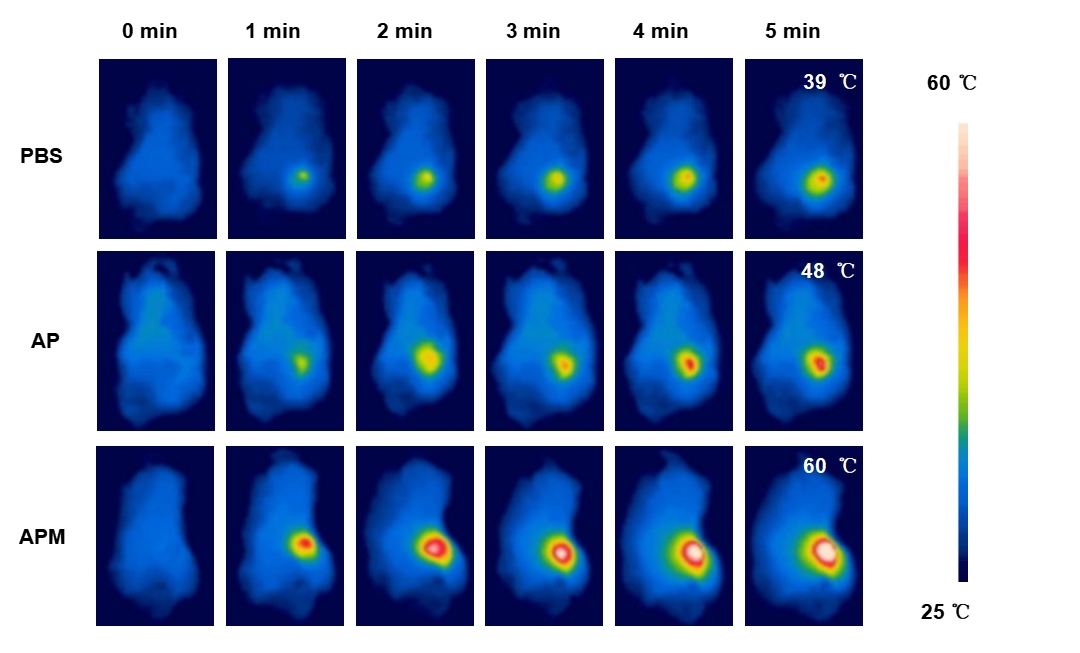


Figure S8. In vivo the photothermal heating images of orthotopic tumor-bearing mice at

24 h post tail injection of APM.


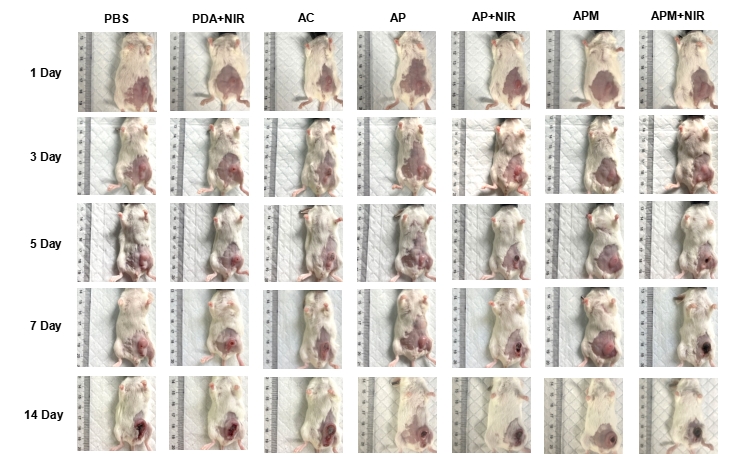


Figure S9. Tumor growth images in orthotopic tumor-bearing mice under various treatments during 14 days. (n=5)


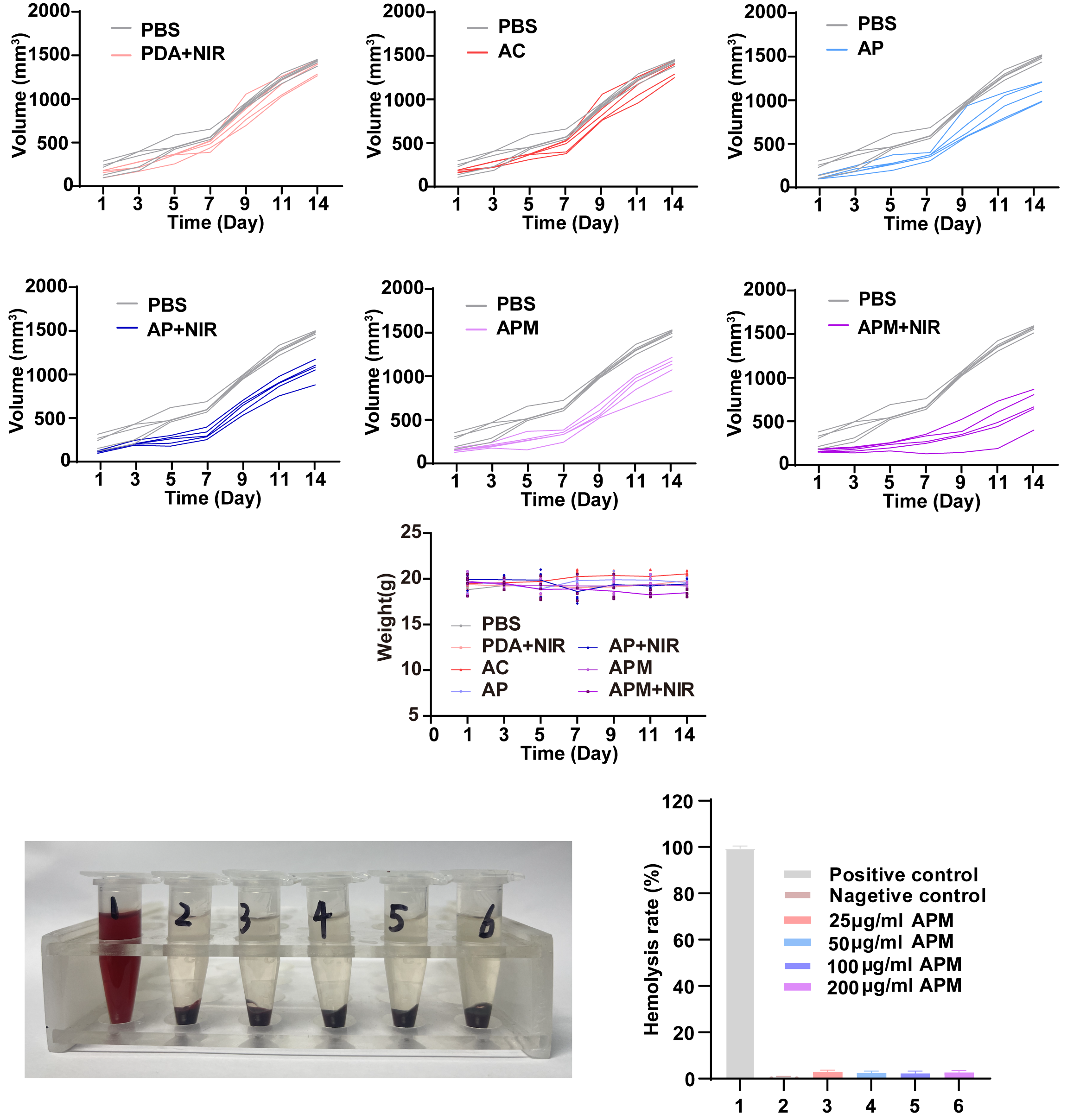


Figure S10. Tumor volume growth curves in orthotopic tumor-bearing mice under various treatments during 14 days. (n=5)


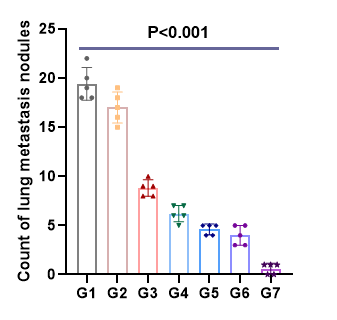


Figure S11. The number of lung metastasis nodules from 4T1 breast tumor-bearing mouses under various treatments. (n=5). (G1: Control, G2: PDA+NIR, G3: AC, G4: AP, G5: AP+NIR, G6: APM, G7: APM+NIR)


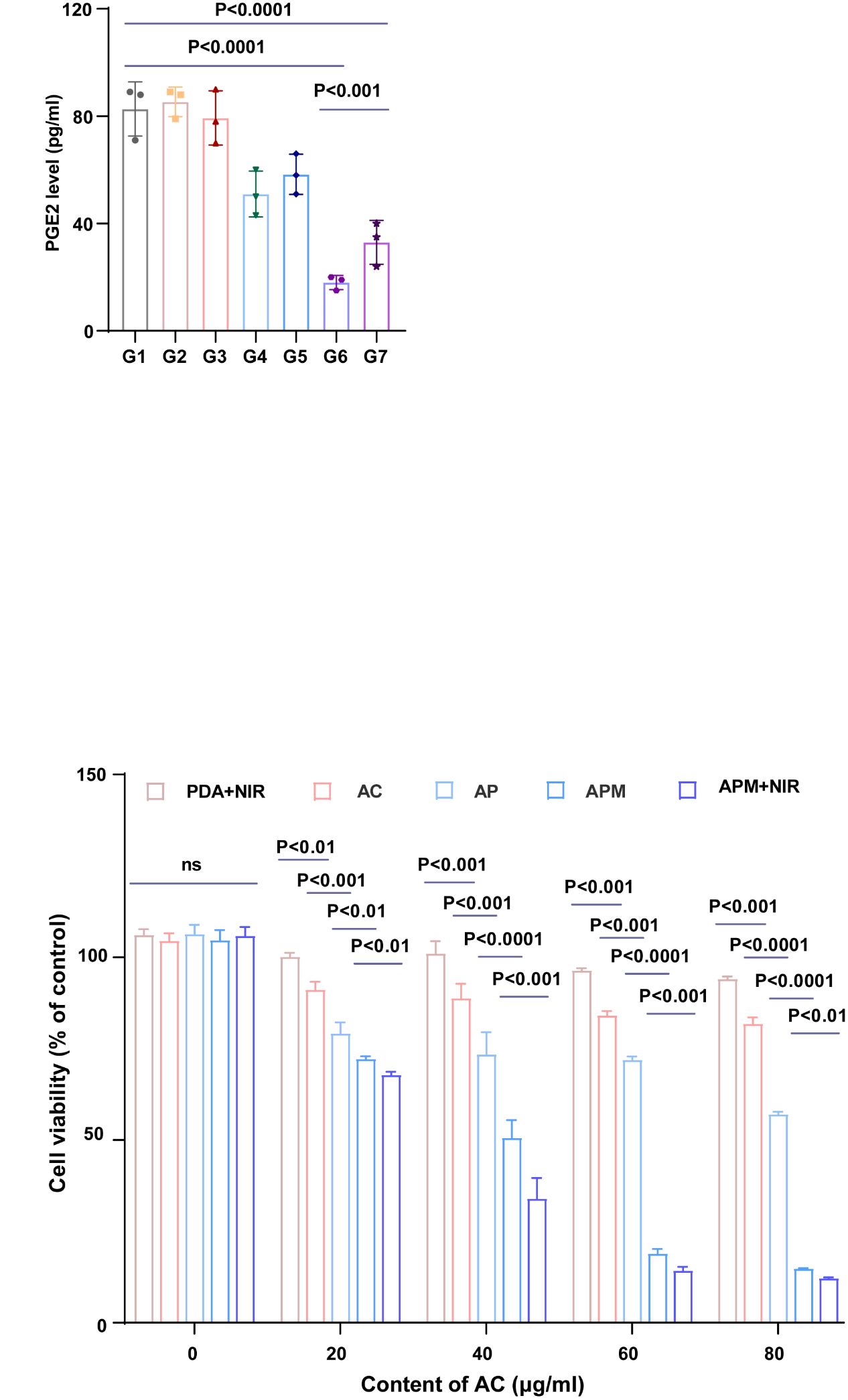


Figure S12. The expression level of PGE2 in tumor issues from orthotopic tumor-bearing mice under various treatments. (n=3). (G1: Control, G2: PDA+NIR, G3: AC, G4: AP, G5: AP+NIR, G6: APM, G7: APM+NIR)


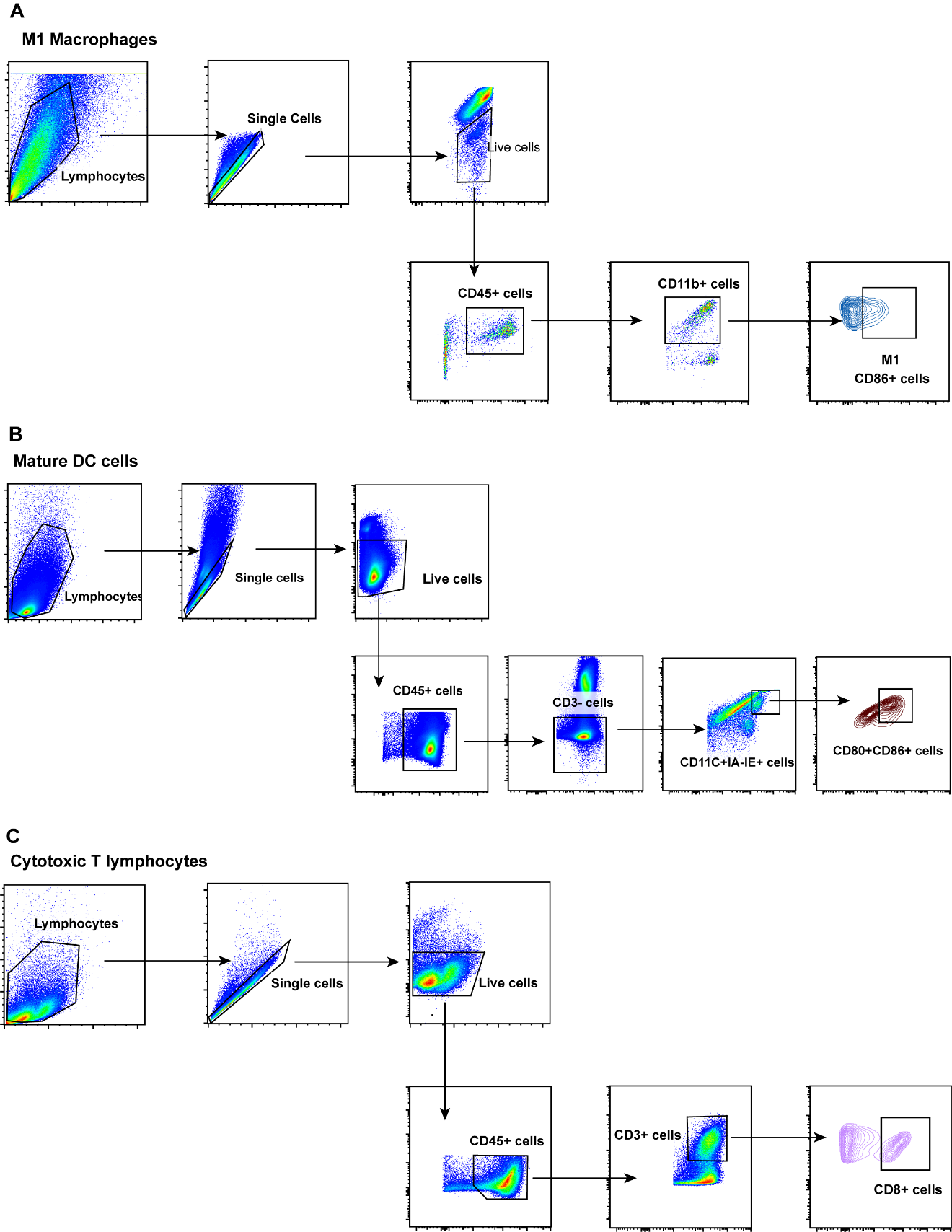


Figure S13. Gating strategy of macrophages, DCs and CTLs. (A) FCM gating strategy of M1 macrophages. (B) FCM gating strategy of mature DCs. (C) FCM gating strategy of cytotoxic T lymphocytes (CTLs).


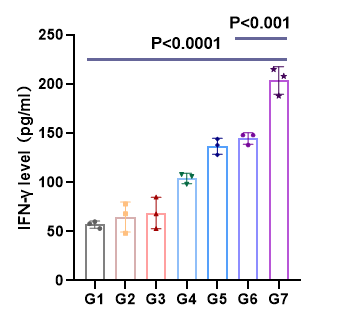


Figure S14. The expression levels of IFN-γ in tumors were analyzed by ELISA under various treatments. (n=3). (G1: Control, G2: PDA+NIR, G3: AC, G4: AP, G5: AP+NIR, G6: APM, G7: APM+NIR)


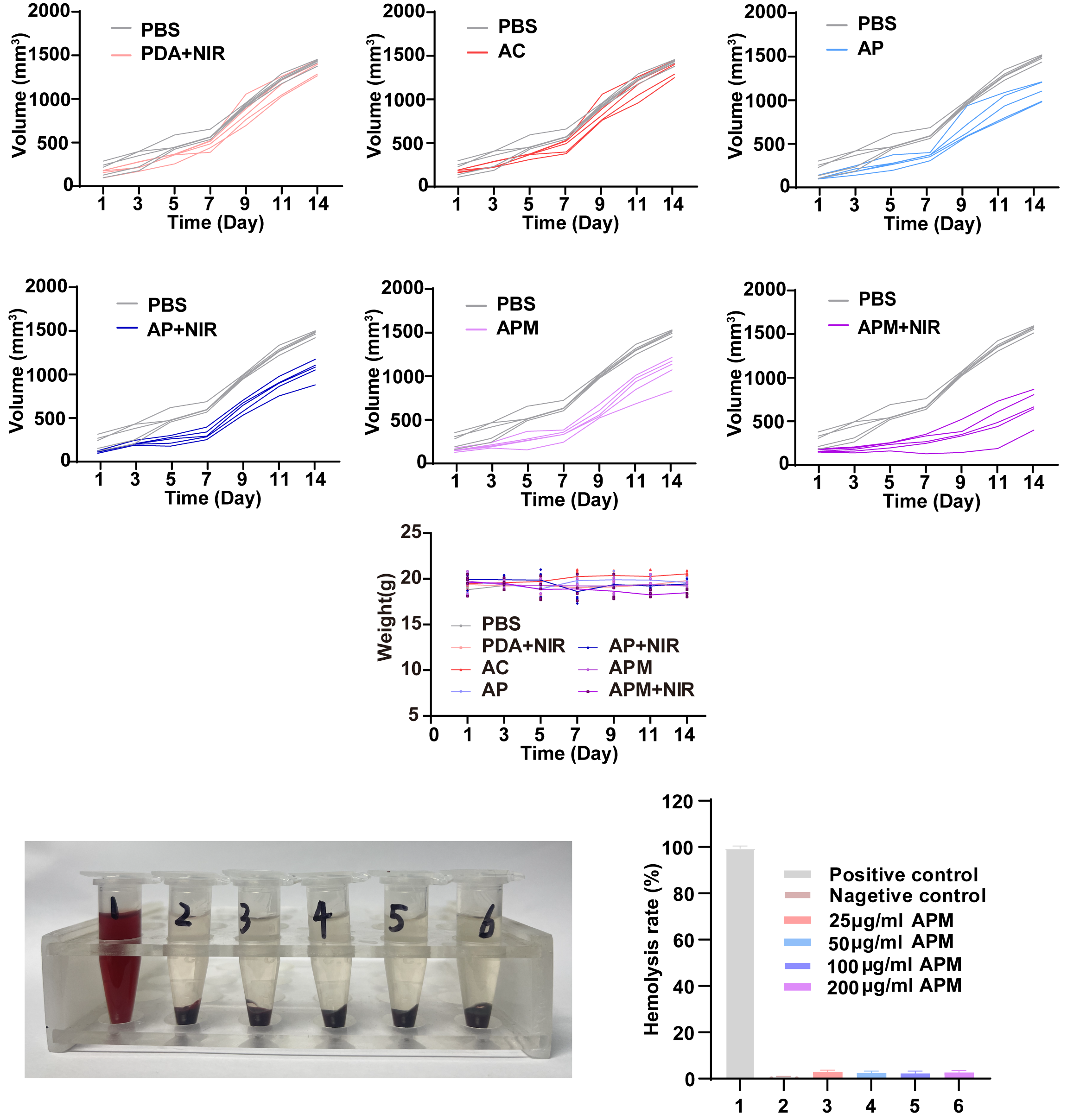


Figure S15. Hemolysis images and hemolysis ratio of APM. (1: Positive control, 2: Negative control, 3: 25 μg/ml APM, 4: 50 μg/ml APM, 3: 100 μg/ml APM, 3: 200 μg/ml APM)


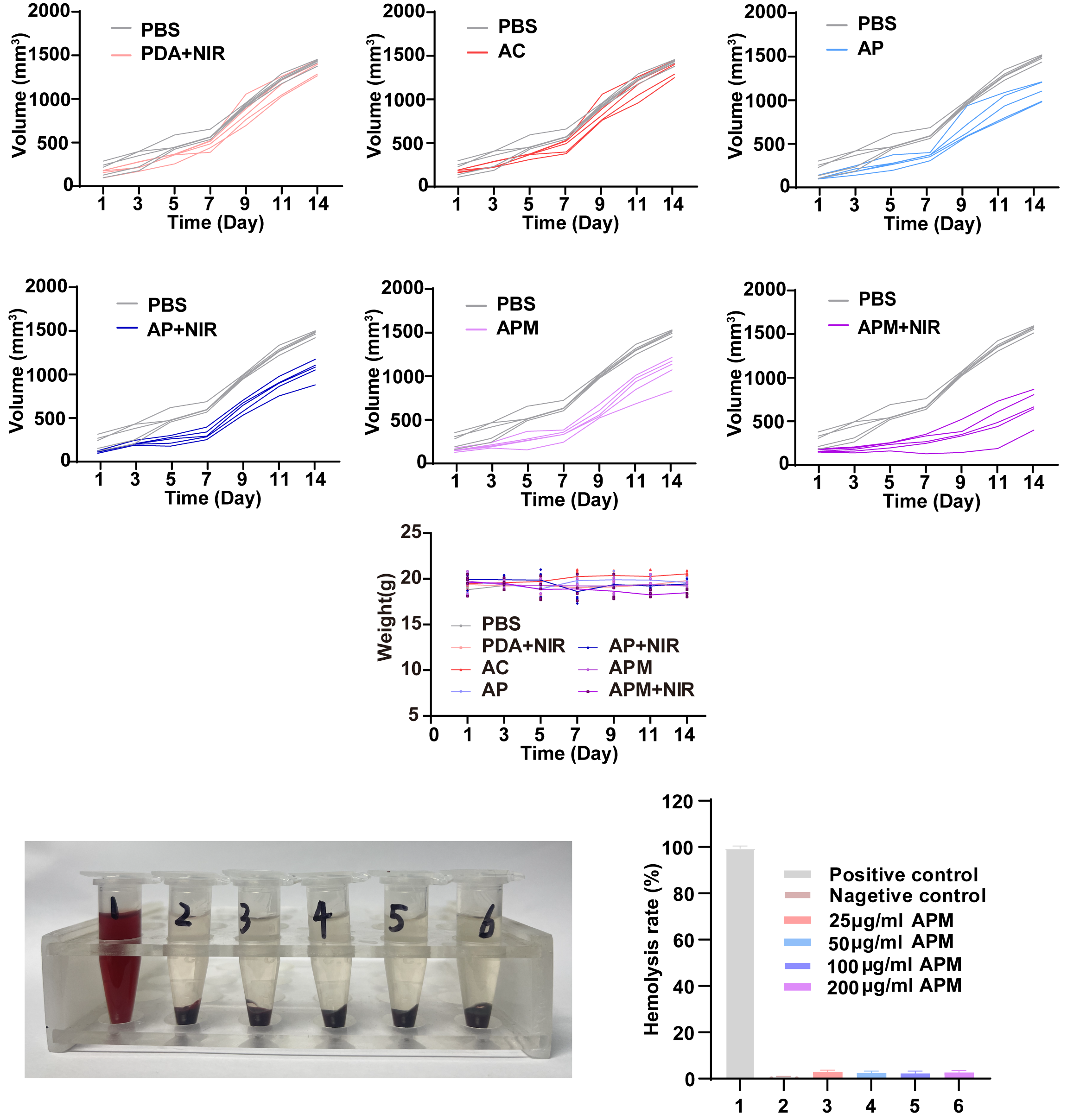


Figure S16. Body weight change curves of orthotopic tumor-bearing mice under various treatments during 14 days. (n=5)

Figure S17. Biochemical blood analysis including expression level of ALT, AST, Urea and Cr in orthotopic tumor-bearing mice under various treatments. (n=5)


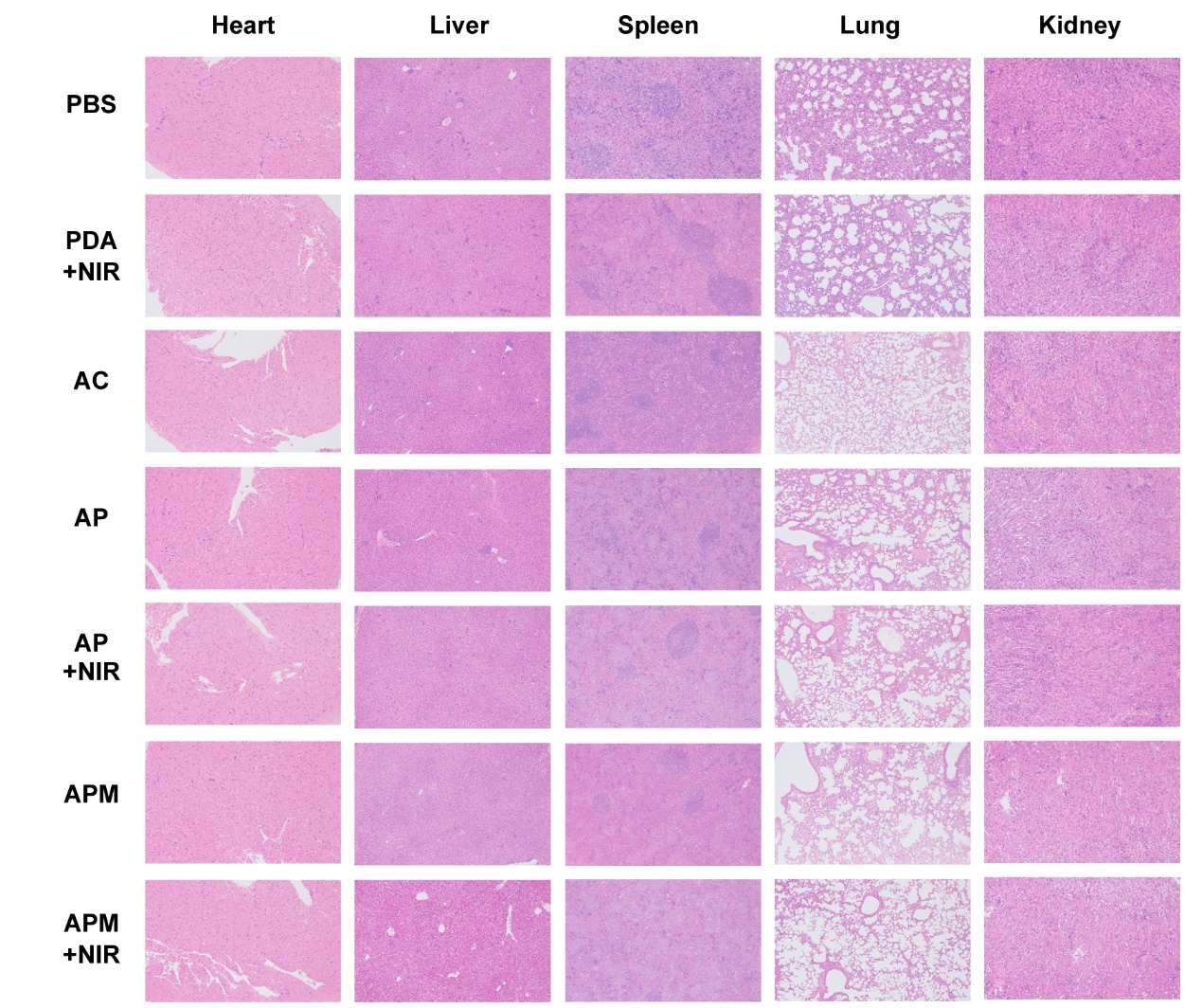


Figure S18. H&E analysis of the major organs including heart, liver, spleen, lung, and kidney in orthotopic tumor-bearing mice under various treatments.
